# US climate policy yields water quality cobenefits in the Mississippi Basin and Gulf of Mexico

**DOI:** 10.1073/pnas.2302087120

**Published:** 2023-10-16

**Authors:** Shan Zuidema, Jing Liu, Maksym G. Chepeliev, David R. Johnson, Uris Lantz C. Baldos, Steve Frolking, Christopher J. Kucharik, Wilfred M. Wollheim, Thomas W. Hertel

**Affiliations:** ^a^Earth Systems Research Center, Institute for the Study of Earth, Oceans, and Space, University of New Hampshire, Durham, NH 03824; ^b^Department of Agricultural Economics, Purdue University, West Lafayette, IN 47907; ^c^Department of Political Science, Purdue University, West Lafayette, IN 47907; ^d^School of Industrial Engineering, Purdue University, West Lafayette, IN 47907; ^e^Department of Plant and Agroecosystem Sciences, University of Wisconsin-Madison, Madison, WI 53706; ^f^Department of Natural Resources and the Environment, University of New Hampshire, Durham, NH 03824

**Keywords:** climate mitigation, carbon price, marine hypoxia, biogeochemistry, agricultural economics

## Abstract

We utilize a coupled economy–agroecology–hydrology modeling framework to capture the cascading impacts of climate change mitigation policy on agriculture and the resulting water quality cobenefits. We analyze a policy that assigns a range of United States government’s social cost of carbon estimates ($51, $76, and $152/ton of CO_2_-equivalents) to fossil fuel–based CO_2_ emissions. This policy raises energy costs and, importantly for agriculture, boosts the price of nitrogen fertilizer production. At the highest carbon price, US carbon emissions are reduced by about 50%, and nitrogen fertilizer prices rise by about 90%, leading to an approximate 15% reduction in fertilizer applications for corn production across the Mississippi River Basin. Corn and soybean production declines by about 7%, increasing crop prices by 6%, while nitrate leaching declines by about 10%. Simulated nitrate export to the Gulf of Mexico decreases by 8%, ultimately shrinking the average midsummer area of the Gulf of Mexico hypoxic area by 3% and hypoxic volume by 4%. We also consider the additional benefits of restored wetlands to mitigate nitrogen loading to reduce hypoxia in the Gulf of Mexico and find a targeted wetland restoration scenario approximately doubles the effect of a low to moderate social cost of carbon. Wetland restoration alone exhibited spillover effects that increased nitrate leaching in other parts of the basin which were mitigated with the inclusion of the carbon policy. We conclude that a national climate policy aimed at reducing greenhouse gas emissions in the United States would have important water quality cobenefits.

## Significance

Reducing climate-warming CO_2_ emissions is a priority for the US public. However, climate mitigation policies can have far-reaching effects. We find evidence of significant cobenefits in terms of the nitrogen cycle, with higher ammonia prices from the pricing of carbon leading to reduced fertilizer application in corn production and diminished nitrate leaching and export to the Gulf of Mexico. Reductions due to a national carbon policy are similar to those resulting from targeted wetland restoration and help to mitigate impacts from crop expansion to new locations that balance crop demand. The decline in N export to the Gulf of Mexico is approximately 10% due to the combination of carbon policy and wetlands mitigation, resulting in a reduction of Gulf hypoxia.

There is a growing imperative to mitigate greenhouse gas emissions in the United States. The United States recently rejoined the Paris Agreement and submitted an updated pledge to reduce net emissions by 50–52% in 2030 relative to 2005 levels (1). The US administration has also developed longer-term climate plans, including achievement of the carbon-free electricity by 2035 and reaching net zero emissions by 2050 (2). In addition, there are numerous state and regional initiatives to reduce emissions (*SI Appendix*, section S.1). In this paper, we explore the consequences of a more comprehensive national climate policy in the United States for crop production and associated environmental impacts, particularly nutrient pollution. A portion of nutrient fertilizer applied to US corn production is not taken up by plants thereby becoming a water pollutant (3–5). Nutrient pollution, mainly nitrate, affects the Gulf of Mexico, where the resulting hypoxia generates a large “dead zone” in most summers (6). Reduction in nitrogen deliveries to the Gulf between 45 and 60% may be necessary to achieve established EPA hypoxia goals (7, 8). However, despite more than a decade of efforts, the five-year average hypoxic zone size is still more than twice the management goal of 5,000 square kilometers by 2035 (9).

**Fig. 1.**
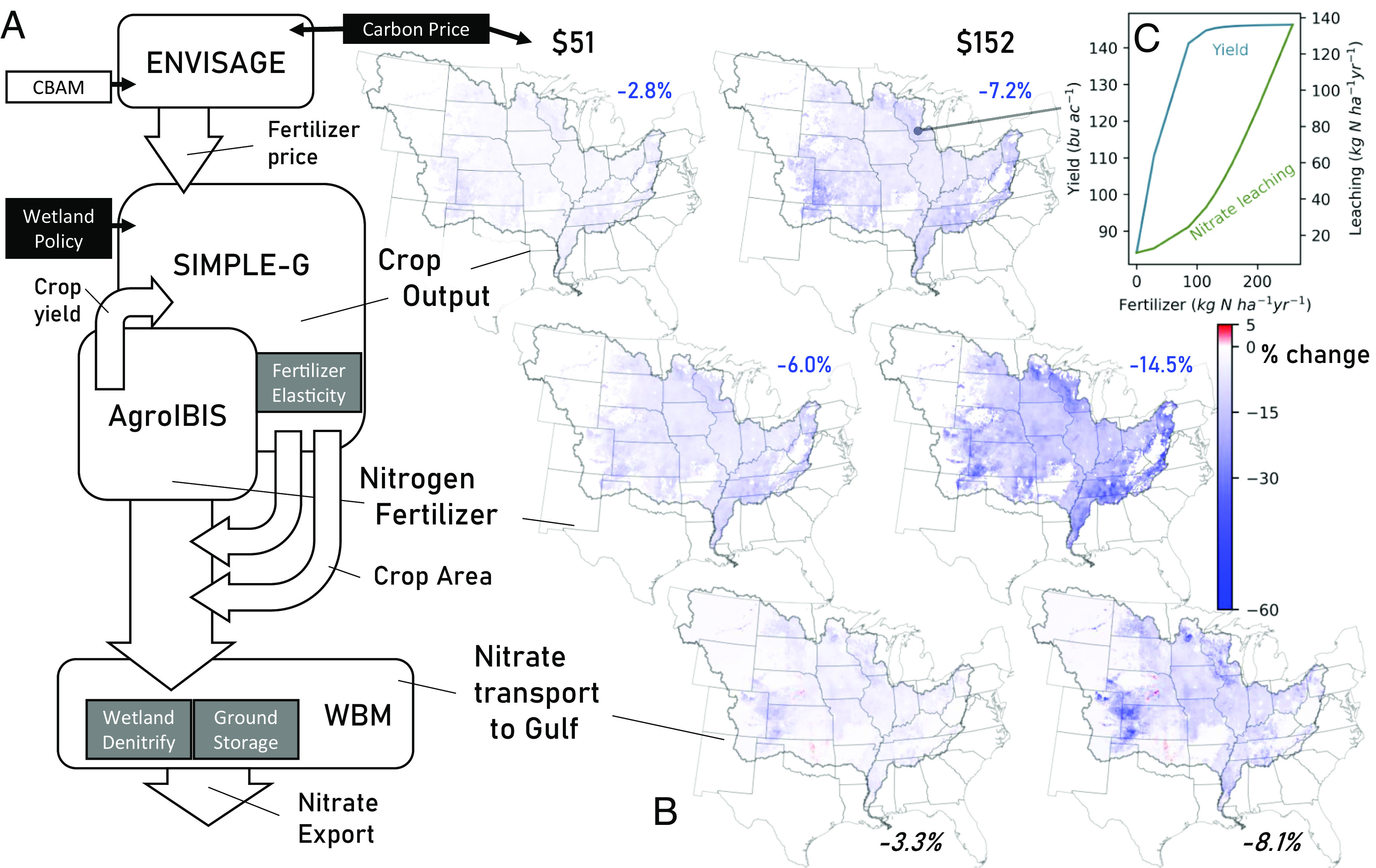
Evaluation using four coupled models to relate carbon pricing to nitrate export to the Gulf of Mexico. (*A*: *Left*) Integrated modeling framework. The ENVISAGE global economic model determines the change in agricultural input prices due to climate policy. These price changes feed into the SIMPLE-G-US-CS gridded economic model of corn–soy production which has been calibrated to capture the yield responses to changes in nitrogen fertilizer applications estimated using the Agro-IBIS agroecosystem model. When confronted with these input price changes, farmers alter their fertilizer usage, thereby changing nitrogen leachate. Nitrogen leachate is routed through the Mississippi River Basin hydrologic system into the Gulf of Mexico using the WBM hydrological model. Imposed policies are depicted in white or black boxes, and uncertainties in key model parameters characterized by sensitivity analyses are listed in gray boxes (Table 1). (*B*: *Right*) Spatial distribution of the relative changes of key factors including crop output (top maps), nitrogen fertilizer applied (*Middle*), and fraction of nitrate flux ultimately entering the Gulf (*Bottom*). Callouts identify points in the modeling framework where each factor is calculated. Inset values for each map provide the basin-wide average value for each depicted variable (blue), and the total change for basin export in black. (*C*: *Top*) Nonlinear response between fertilizer application rate with crop yield and nitrate leachate below the root zone represented, respectively, by Gompertz and quadratic functions. Callout identifies the grid cell location of this example response.

Here, we investigate the potential cobenefits of a national climate policy that would alter the relative prices of carbon-intensive products, thereby altering farming practices and their associated environmental impacts. We utilize a coupled economy–agroecology–hydrology modeling framework to evaluate the effect of climate mitigation on US agriculture and uncover potential water quality cobenefits (Fig. 1). We focus on a climate policy that entails pricing fossil fuel-based combustion and process-based CO_2_ emissions using the US government’s social cost of carbon, ranging from $0 to $152/ton. A global computable general equilibrium model, ENVISAGE, estimated changing costs of crop inputs by imposing the social cost of carbon on US emissions which serve as inputs to a spatially resolved partial equilibrium model of agroeconomic activity (SIMPLE-G-US-CS). The agroecological model (Agro-IBIS) used the resulting changes in fertilization rates and land cover to estimate nitrate leaching, which provided boundary conditions to a gridded, watershed-scale model of hydrology and nutrient processing (WBM), ultimately permitting the estimation of nitrate export from the Mississippi River watershed to the Gulf of Mexico. Results are presented as basin-wide changes of each output relative to a model scenario with no carbon pricing mechanism. We stratify our simulations across four estimates of the social cost of carbon and vary key parameters across the suite of models to provide an indication of model sensitivities. We further integrate our analyses with scenarios of wetland restoration to evaluate the possible interactions between multiple strategies for reducing nitrate pollution.

## Results

### Climate Policy Can Result in Significant Reductions in Nitrate Leaching across the Mississippi River Basin.

Climate mitigation policy raises the cost of carbon-intensive products across the entire economy. As a result, US carbon emissions decline by 29% to 50%; these reductions represent 4.6% to 8.0% of global carbon emissions and cover the range of reductions specified by the Paris Accord (1). Given the intensity of natural gas use in the ammonia fertilizer industry for both combustion and process-based emissions, fertilizer prices rise sharply (nearly doubling prices under the $152 carbon price). Furthermore, by assuming implementation of a Carbon Border Adjustment Mechanism in conjunction with the climate policy, we minimize any advantage to importing nitrogen fertilizer from other regions with less stringent climate policies (10).

A permanent price hike based on the $152 carbon pricing scenario results in a significant drop (13.3 to 16.0%) in fertilizer applications on land predominantly planted with corn–soy rotations across the basin. Fertilizer reductions occur through a combination of reduced planting of corn and soy (*SI Appendix*, Table S4) and reduced fertilization rates of all crops (*SI Appendix*, Table S5). At the largest price increase, the total corn–soy area in production declines by about 2% (10,000 km^2^), where we assume that corn and soy croplands revert to prior land covers (primarily cultivated and uncultivated grassland). Reductions in crop area explain between 10 to 20% of the reduction in nitrate leachate from corn and soy cropland, whereas fertilization rate reductions explain the majority of leachate reduction. Previous fertilizer price increases caused similar reductions in fertilizer applications (11) with a similar balance of crop area and fertilizer rate reductions (12).

**Fig. 2.**
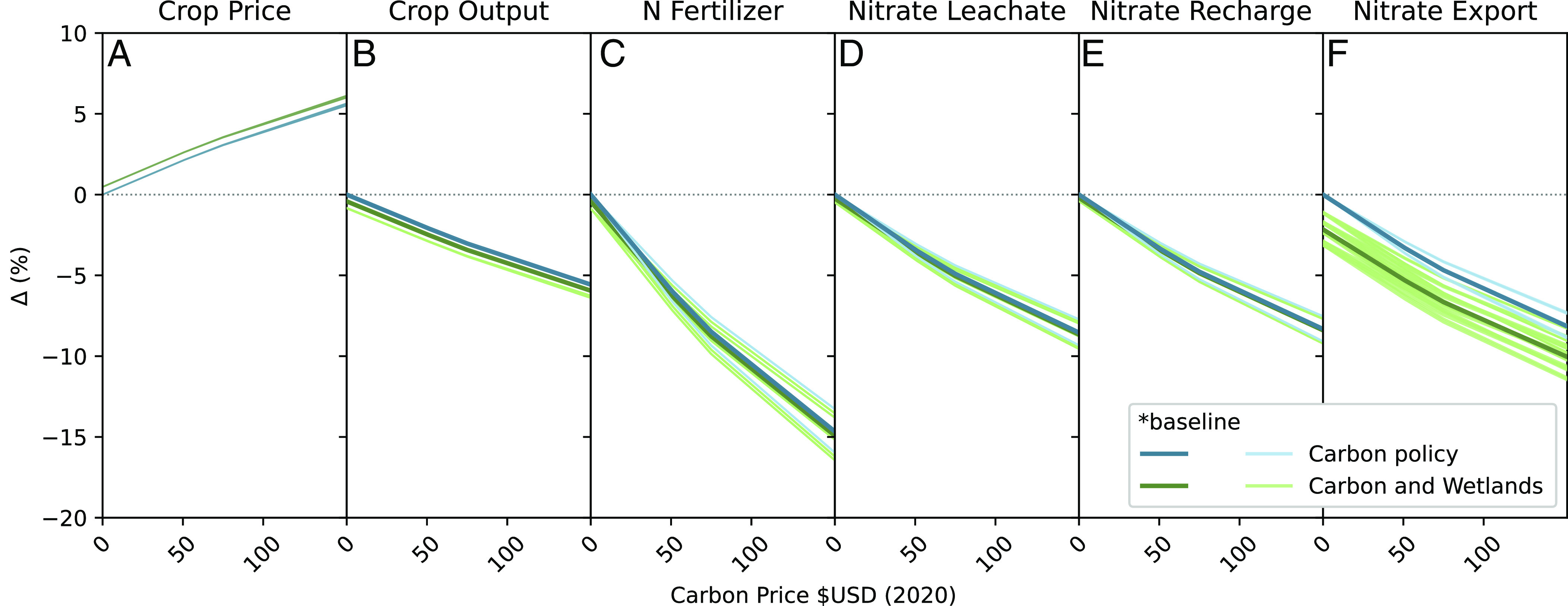
Impacts of an increasingly stringent climate policy, with and without wetlands restoration. The panels display the impacts on (*A*) corn–soy crop price, (*B*) crop output, (*C*) nitrate fertilizer application, (*D*) nitrate leaching, (*E*) nitrate in groundwater recharge, and (*F*) nitrate export to the Gulf of Mexico. The carbon price (*x*-axis) varies from $0 to $152/ton CO_2_-e. Separate traces represent different policy scenarios and realizations of uncertain system parameters (Table 1). Simulations using baseline parameter values in SIMPLE-G and WBM (asterisk in Table 1) are in bold. For variables further to the right, additional human and natural processes are simulated, and the effects of parameter sensitivity become more evident. In each panel, traces formed from all 174 simulations are displayed; however, insensitivity of variables to some parameters (e.g., crop price is unaffected by deep groundwater storage) result in traces that are colinear.

Our results are obtained from a suite of parameterizations that provide context from key uncertainties described in more detail below. The combination of reduced crop area and decreased fertilization rates results in about 6% decline in crop output in the Mississippi watershed and a commensurate rise in corn–soy prices (Fig. 2) relative to a scenario with no carbon price. Nitrate leaching from natural vegetation is unaffected by changes in the fertilizer price and crops other than corn/soy are less affected, so the relative changes in total watershed leachate below the rootzone (8 to 9%) are smaller than the changes in fertilization (Fig. 2).

The reduction in on-farm (local) nitrate leaching translates into less nitrate percolating to groundwater through recharge and lower concentrations delivered to streams and rivers. However, because some existing nitrate leachate is mitigated through natural denitrification and long-term storage in soils and groundwater, as well as ambient wetland, stream, and reservoir denitrification, relatively less reduction in export to the Gulf of Mexico is observed, from 7 to almost 9% relative reduction for the $152 carbon price scenario (Figs. 1 and 2). This represents a significant cobenefit to an action that would meet existing obligations for carbon reduction.

### Relative Impact and Interactions of Climate Policy and Wetland Restoration for Reducing Nitrate Export.

Nitrogen mitigation is not the purpose of climate policy but indirectly leads to nitrate export reduction. Interventions that target mitigation of nitrate exports associated with cultivation emphasize wetland restoration or construction (13). To assess the relative impact and interactions of the carbon policy described above with wetland interventions, we implement scenarios assuming adoption of wetland construction following the federal Farmable Wetland Program (FWP) (14). Wetland scenarios followed guidance for efficient land conversion to maximize nitrate reduction by targeting tile-drained corn and soy and to minimize declines in crop production by utilizing large wetland catchments (15, 16) (*SI Appendix*). A total of 4,500 km^2^ of crops were retired, with 1,000 km^2^ new active wetland areas constructed, while reserving the remaining 3,500 km^2^ as uncropped wetland buffer. These new wetlands received runoff from 206,000 km^2^ of tile-drained corn and soy crops, representing 24% of all cropland and 40% of nitrate runoff in the basin.

We estimate that the full adoption of FWP as a stand-alone scenario could reduce nitrate export to the Gulf by up to 3.0%, which is comparable to the 3.0 to 3.6% reduction that resulted from the $51 carbon price scenario alone. However, wetlands achieved similar nitrate reduction by removing only one-fifth as much land from corn/soy production that SIMPLE-G-CS-US predicts at the $51 carbon price. The wetlands in the FWP scenarios remove much less than the 40% nitrate leachate they intercept for several reasons. First, wetland restoration alone induces crop production in new areas as predicted by SIMPLE-G-CS-US (see below) with a minimal net effect on basin-wide crop output (−0.6%), price (+0.5%), fertilizer applications (−0.9%), and nitrate leachate (−0.5%) (Fig. 2 *A* and *B*). Second, as an edge-of-field policy, wetland restoration has a negligible effect on leaching out of the root zone (Fig. 2*D*). Rather, nitrate is removed following transport to the wetland, resulting in declining nitrate exports (Fig. 2*F*). However, nitrate removal capacity in wetlands varies seasonally and during storms due to changes in temperature and residence times (17), reducing their impact on nitrate reaching streams. Finally, the model assumes a large fraction of leachate bypasses wetlands to the subsurface to reflect the long-term storage of agricultural nitrate in groundwater (18) and is unavailable for treatment by constructed wetlands. The magnitude of nitrate export reduction from adoption of wetland restoration is comparable to the effects estimated from integrated assessment modeling (16, 19), but significantly less than estimated solely from physical potential (20).

The mitigation curves for CO_2_ pricing alone and those combined with wetland restoration are nearly parallel, indicating that wetland restoration is roughly additive with the carbon policy (Fig. 2*F*). This linearity occurs because the influences of the two programs are spatially complementary. While the FWP emphasizes reduction of agricultural nitrate from the most productive areas of the Corn Belt, the carbon policy would likely result in declines of marginal agricultural lands due to increased costs of farming under higher energy and fertilizer prices.

### Climate Policy Reduces Adverse Spillover Effects from Wetland Mitigation Program Alone.

**Fig. 3.**
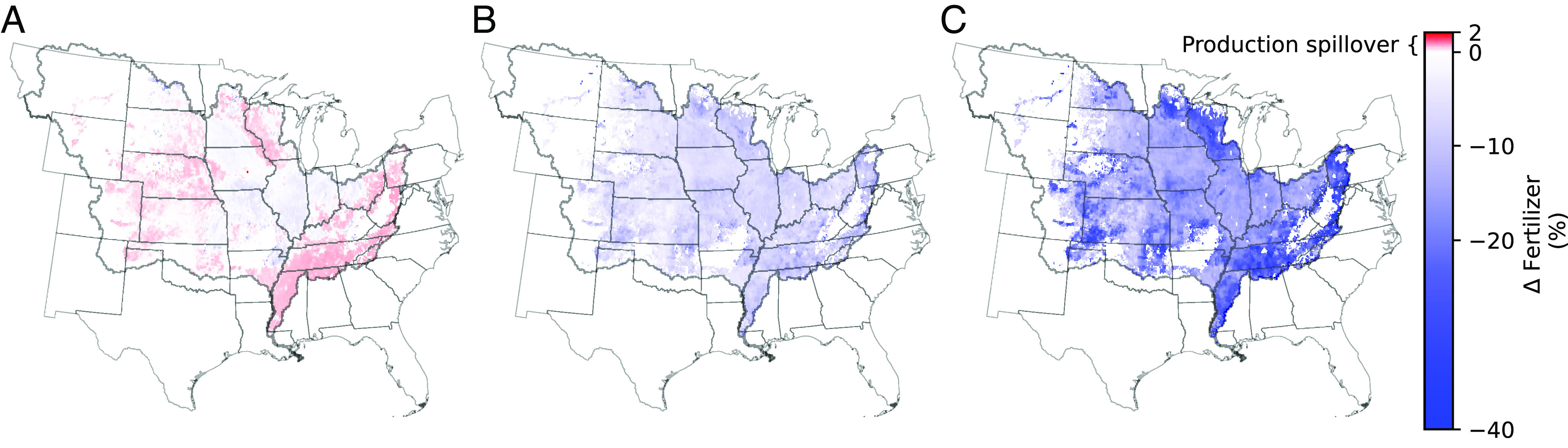
Effect on nitrogen fertilizer applications of coupling wetland restoration with carbon pricing. (*A*) Absent carbon pricing ($0), restoration of wetlands over the tile-drained land causes market-mediated production spillovers of cropland beyond the central Corn Belt, thereby increasing local fertilizer use across much of the MRB and offsetting the impacts of the wetland restoration policy. Imposing carbon pricing nearly eliminates this spillover effect and decreases fertilizer applications over most of the Mississippi Basin at (*B*) $51 and (*C*) $152 carbon prices.

In the absence of an effective climate policy (i.e., $0/ton CO_2_-e carbon price), wetland restoration results in increased leaching in agricultural areas with no wetlands, thereby limiting the effectiveness of the wetland-only scenario (Fig. 3*A*). This is a direct consequence of the market-mediated spillover effect of an intervention that targets only a portion of the corn–soy production in tile-drained areas (21, 22). In major corn- and soybean-producing states in the upper Midwest, land is taken out of production for wetland restoration (Fig. 3*A*). As resulting crop prices rise and the market price of nitrogen fertilizer declines in response to this intervention, the agroeconomic dynamics in SIMPLE-G-US-CS restore supply–demand balances by increasing crop production and fertilizer applications elsewhere (Fig. 3*A*). As a result, the total land producing corn and soy in the MRB is on net reduced by only about 700 km^2^ despite 4,500 km^2^ of land being taken out of production under the full wetland adoption scenario. In terms of new crop area, additional spillover occurs outside the MRB—half as much as occurs within MRB. In addition, there are international cropland area spillovers amounting to three times the MRB spillover effects owing to lower yields overseas. Nitrate delivery to the Gulf of Mexico from wetland restoration would have been reduced by an additional 18% without these spillovers. This spillover-induced shifting of leachate locations and the ensuing expansion of production into marginal lands are both significant impacts from a policy perspective, illustrating the challenge posed by a program that treats only the tile-drained portion of the area contributing to the Mississippi Basin nitrate leaching problem, and have not been previously considered by wetland restoration impact studies. In contrast, the carbon pricing policy discourages fertilizer applications across the entire Mississippi Basin and largely eliminates the negative spillover effects created by the wetlands-only scenario (Fig. 3 *B* and *C*). Furthermore, throughout the range of tested carbon prices, the nitrate removal from wetlands remained nearly linear because any reduction in the absolute amount of nitrate entering wetlands from surrounding croplands would result in a higher proportion retained due to increased efficiency of the kinetics of wetland denitrification (*Materials and Methods*).

### Climate Policy Can Contribute to Improved Ecological Outcomes in the Gulf of Mexico.

**Fig. 4.**
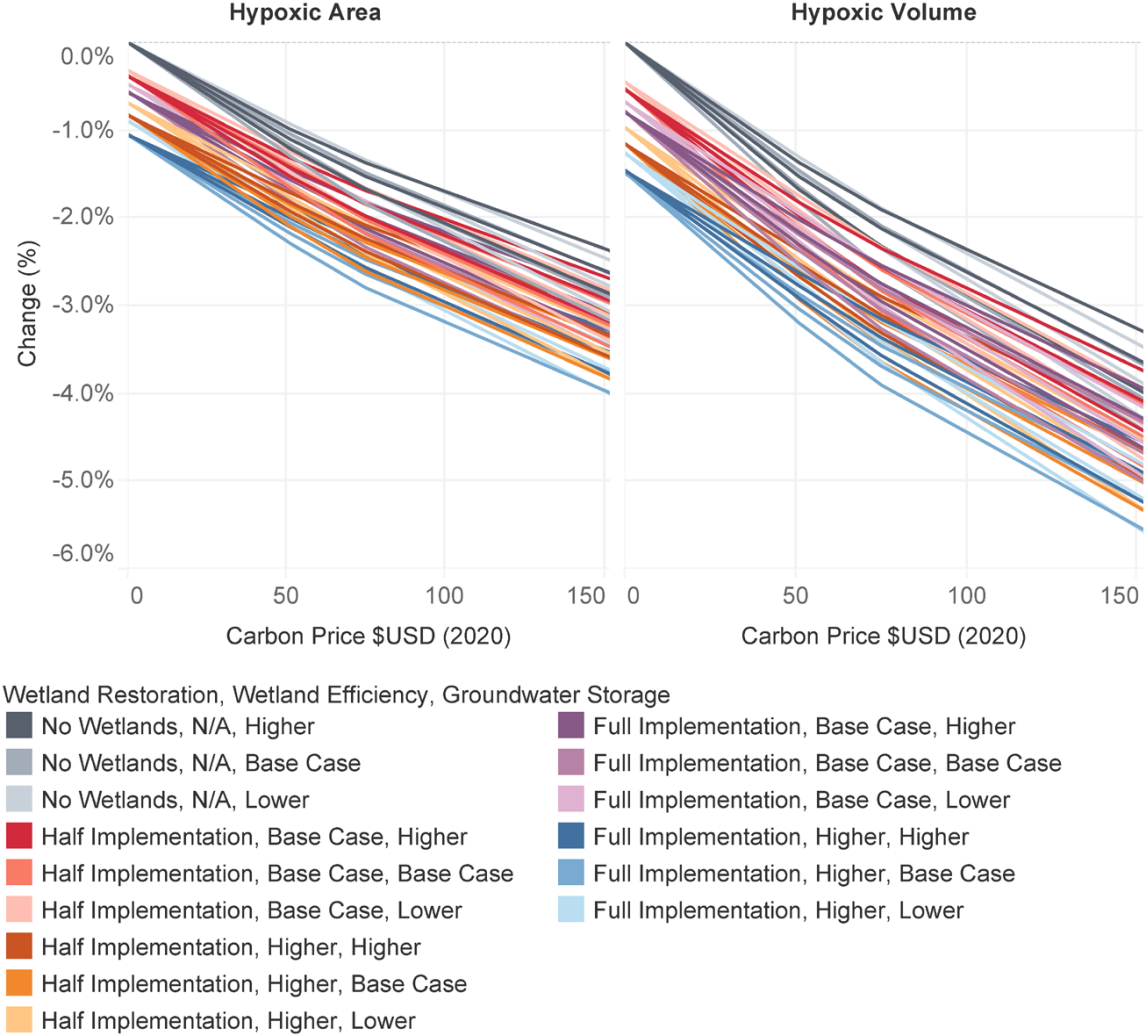
Change in average peak hypoxic area (*Left*) and volume (*Right*) in the northern Gulf of Mexico. Traces represent different combinations of uncertain model parameters (Table 1): cases without wetland restoration (gray), full implementation of wetlands with base assumptions for wetland denitrification efficiency (purple), full implementation with higher efficiency (blue), half implementation with base efficiency (red), and half implementation with higher efficiency (orange). Darker hues indicate greater groundwater storage. Hypoxia area and volume impacts are based on simulated total N delivery to Gulf (7).

The reduced export of nitrogen to the Gulf of Mexico translates into a reduction of the maximum area and volume of hypoxic water, defined as a dissolved oxygen concentration in bottom waters of less than 2 mg L^−1^. From prior modeling of hypoxic zone response (23), a 1% decrease in nitrogen flux leads to a decrease in peak annual hypoxic area of approximately 0.4% and in volume of 0.6%. The decreases are proportionally smaller than the decline in nitrate exports (Fig. 4) as other nitrogen forms remain unchanged by the interventions which target inorganic nitrogen (*SI Appendix*). At the carbon-pricing levels we simulated, peak annual hypoxic area could be reduced by up to 3.2% and volume by up to 4.4% relative to a scenario with no carbon pricing. By including the full adoption of FWP wetlands, hypoxic area could be reduced by up to 4.0% and hypoxic volume by up to 5.6%. We note that changes to the coastal ecosystem, such as planned Mississippi River diversions, could alter this relationship. Some researchers have found recent increases in the size of hypoxic zones for the same amount of nitrate loading; warming temperatures and increased frequency of intense precipitation under climate change could increase nutrient runoff and produce stronger stratification gradients in the northern Gulf (6).

## Discussion

Our analysis strongly suggests that implementing climate policies such as carbon pricing generates significant environmental cobenefits. Seasonal hypoxia in the Gulf of Mexico dates back to the 1950’s. Since then, nitrogen loading has steadily grown and the seasonal hypoxia area now often exceeds 20,000 km^2^, despite many government programs aimed at curbing agricultural runoff (6, 24–27). By pricing carbon emissions from fossil fuels and implementing a carbon border adjustment to maintain domestic fertilizer industry competitiveness, we anticipate a strong increase in the price of nitrogen fertilizer, thereby reducing fertilizer applications across the Mississippi Basin. The net result is a relative reduction in nitrate export to the Gulf of about 8%, and a potential reduction in the hypoxic volume of approximately 4%, under the most ambitious carbon price we explored ($152/ton CO_2_-e). Although not the goal of the climate mitigation policy imposed here, its unintended impact on reduced nitrate delivery to the Gulf is significant and potentially larger than other policies that directly address the Gulf hypoxia issue such as wetland restoration (16). The potential for synergistic benefits we see here is comparable to win–win outcomes among multiple ecosystem services in well-managed socio-ecological systems (28).

There is considerable uncertainty at each stage of this integrated modeling framework that has the potential to cascade through the entire system. A full quantification of these compound uncertainties is beyond the scope of this paper. However, we give an idea of these cascading uncertainties by varying key parameters of each model (Table 1) and investigating how they interact. Generally, the compound uncertainties behave supra-linearly as inputs cascade through the models. For instance, varying the elasticity of nitrogen fertilizer in corn production tends to have a much greater effect on fertilizer application, crop leachate, recharge, export (Fig. 2 *C*–*F*), hypoxic area, and hypoxic volume variables (Fig. 4) than it has on the directly associated variables of crop price and crop output (Fig. 2 *A* and *B*). Therefore, there is potential for uncharacterized uncertainties in the coupled models to similarly influence biophysical predictions. Nevertheless, this sensitivity analysis reveals that our key finding that nitrate export and measures of hypoxia decline monotonically with increasing carbon price is robust to the cascading uncertainties. In particular, the combination of climate policy and wetland construction in the mitigation of nitrate export to the Gulf remains approximately additive throughout the range of tested parameter uncertainties.

**Table 1.** Key model parameter values used for 174 simulation scenarios to assess cascading uncertainties

Source of Uncertainty (*MODEL*, Units)	Parameter settings			
	Absent	Low	Medium	High
Carbon Price (*ENVISAGE*, $US)	0^*^	51	76	152
Wetland Restoration (*SIMPLE-G* & *WBM*, Percent)	0^*^	—	50	100
Elasticity of substitution^†^ Irrigated Rainfed (*SIMPLE-G*, Dimensionless)	—	0.18 0.13	0.24^*^ 0.18^*^	0.30 0.22
Deep groundwater storage^‡^ (*WBM*, Fraction)	—	0.53	0.59^*^	0.65
Denitrification uptake efficiency^§^ (*WBM*, m/year)	—	—	27^*^	54

^†^Denotes the Basin-wide fertilizer-weighted average.

^‡^Fraction of nitrate percolating to long-term subsurface storage (29).

^§^Uptake velocity of denitrification at an ambient nitrate concentration equal to 1 mg L^−1^ (30).

^*^Denotes baseline values.

Perhaps the greatest source of uncertainty rests with the climate policy itself. Our framework did not provide a comprehensive analysis of emissions reduction of non-CO_2_ greenhouse gases. The impact on N_2_O emissions in particular should be explored further, as they are closely tied to the fate of nitrate (31, 32). While we have varied the carbon price across a wide range of scenarios, ranging from $0 to $152/ton CO_2_-e, climate policies in the US have hitherto been piecemeal and often temporary, depending on the political party in power (33, 34). Furthermore, to date, the most aggressive climate actions have been taken at the state and local levels, resulting in a patchwork of regulations which are often very difficult to quantify, much less incorporate into a national economic analysis (35, 36). For this reason, these are not predictions of what is most likely to happen, but rather insights into the potential interactions between a comprehensive climate policy, agricultural activity, and natural ecosystem services (37). In addition, it should be stressed that our analysis is based on an ensemble of modeling tools focused on a medium-term time horizon. In this regard, consideration of the long-term evolution of the economic, energy, and agricultural systems, including representation of alternative technologies and mitigation options that are currently under development, would be an important extension of the assessment presented here.

The cobenefits of the carbon pricing policy to mitigate groundwater contamination by nitrate, estimated to result in an 8% reduction in nitrate flux to groundwater, stands to improve health outcomes of vulnerable communities. Presently nitrate contamination of groundwater is three times more likely to adversely impact communities with a high proportion of residents identified as Hispanic by the Census Bureau (3), as well as rural communities using self-supplied groundwater for home use (38). Policies that mitigate groundwater contamination by nitrate therefore stand to improve health outcomes of underserved groups. The carbon prices imposed in this study resulted in consequential reductions on the order of 7.2% in corn and soy output from the Mississippi River Basin—roughly 20,000 tons of corn-equivalent output. SIMPLE-G-US-CS accommodated reduced corn and soy production through reduced domestic use (primarily in livestock feed), as well as diminished grain exports. In response to the elevated corn–soy prices, foreign producers would expand production. The environmental and health impacts of moving this production outside of the US depend on the agricultural and ecosystem contexts of where this production moves and therefore warrant further examination (39). The full suite of consequences of the carbon policy is not considered here but should aim to distribute net benefits as equitably as possible.

The revenues generated through carbon pricing policies can be recycled using alternative mechanisms. Here, we assumed that households are the beneficiaries of this revenue, and we do not account for any potential direct changes to agricultural policies. Considering the environmental cobenefits that mitigate hypoxia in the Gulf, it is reasonable to consider alternative uses of this revenue that could more completely address water quality concerns (40). For instance, the adoption of wetland restoration practices, despite the positive environmental benefits, has been slow in part due to cost (41–43). While we estimate the direct impacts on nitrate reduction to be modest and in line with other studies of edge-of-field wetland restoration (19, 44), their environmental benefits are proportionally much higher than any impacts on production or price (Fig. 2). Across different wetland mitigation strategies, the nitrate reductions fall short of the 45% to 60% cuts necessary for ecosystem health (7, 25). Because edge-of-field restoration strategies such as those simulated here would not likely achieve needed nitrate reduction, alternative and multiple strategies are required to reduce nitrate export sufficiently to protect the marine ecosystem in the Gulf of Mexico (16, 19, 45). The revenue generated by a carbon policy could be used to support such additional reduction strategies.

A nationwide carbon policy would not only allow the United States to begin to reach its carbon emission targets but would have the added, but unintended, benefit of reducing nitrogen exports to the Gulf of Mexico. While by itself the carbon policy would not be sufficient to meet mitigation targets, it represents almost 20% of the required N export reduction. This indirect effect is of a similar or greater magnitude to some of the management plans directly targeting N exports, such as the wetland restoration. Carbon pricing therefore represents another potential tool for policymakers and managers to alleviate a problem, the hypoxic zone of the Gulf of Mexico, that has not been solved despite decades of planning and effort. Comprehensive, system-wide analyses such as the coupled economic–agricultural–biogeophysical modeling framework developed here offer the potential to identify other pathways by which to improve management of multiple, interacting ecosystem services at broad spatial scales.

## Materials and Methods

Our analysis relates biophysical outcomes of nitrate pollution reduction in the Mississippi River Basin to broad-scale policy decisions using a coupled modeling framework relating global economic activity (*ENVISAGE*) to US agroeconomic activity (*SIMPLE-G-US-CS*), the resulting agronomic outcomes (*Agro-IBIS*), watershed-scale nitrate transport and environmental processing (*WBM*), and ultimately to expected changes to marine hypoxic area and volume in the Gulf of Mexico. Here, we describe each of the models, our hypoxia estimation, and experimental design. Additional details regarding model calculations are available as *SI Appendix*.

### *ENVISAGE*.

We begin our analysis by running a static version of *ENVISAGE* global computable general equilibrium (CGE) model (46). The ENVISAGE model used in this study is calibrated to the Global Trade Analysis Project (GTAP) 10 Power Data Base with 2014 reference year, which distinguishes 141 regions and 76 sectors (47). The latter includes 11 electricity generation technologies, as well as an electricity transmission and distribution activity. This is the most up-to-date version of the GTAP-Power Data Base. For the purposes of this paper, we use an aggregation that includes 14 regions (*SI Appendix*, Table S1) and 41 activities (*SI Appendix*, Table S2). The main strength of a CGE modelling framework, like ENVISAGE, is the consistent representation of the interdependencies between different sectors, agents, and markets in the global economy. By capturing both the supply and demand sides, the model represents adjustments in quantities and prices following the implementation of a policy shock. For instance, if carbon pricing is implemented in the model, this leads to increasing prices energy, reducing energy supply and demand, as well as corresponding shifts in the energy supply mix, with increasing share of low-carbon technologies. Additional details on the choice of the model aggregation and source of the key model parameters are provided in *SI Appendix*, section S.2.

Our study simulated four different mitigation scenarios each involving a different social cost of carbon: $US 0, 51, 76, and 152/ton CO_2_-e, reflecting the underlying uncertainty in the appropriate social discount rate as well as in the science of climate impacts (48). This climate policy action is implemented in the United States only, with the analysis assuming prior implementation of a comparable policy in the European Union.Importantly, we simultaneously implement a Carbon Border Adjustment Mechanism to protect domestic industries, avoid carbon leakage, and prevent the importation of additional carbon-intensive products (e.g., fertilizer) from countries without a comparable climate policy (*SI Appendix*, section S.3.1). The carbon-pricing policy raises energy costs across the economy, with natural gas costs rising sharply. Importantly for agriculture, the rise in natural gas costs boosts the price of ammonia, a key ingredient in nitrogen fertilizer. The consequences for the full range of agricultural input prices are a key output from the *ENVISAGE* model for this integrated analysis (Fig. 1). Additional details on the carbon pricing scenario framework are provided in *SI Appendix*, section S.3.1. A key outcome of the ENVISAGE model that impacts the decision-making process in the agricultural sector is the change in the price of ammonia fertilizer. The assumptions behind ammonia production cost structure are based on the conventional (fossil-based) ammonia production process and are discussed in *SI Appendix*, section S.3.2. Considering the medium-term analysis timeframe in this study, we do not consider alternative ammonia production technologies that are based on the combination of electrolysis and renewable energy sources, which are expected to become economically viable in the post-2030 period (49). *SI Appendix*, section S.3.3 discusses implications of the carbon pricing scenarios considered here in the context of the future ammonia production using electrolysis.

### *SIMPLE-G-US-CS*.

We translate the national impacts of climate policy produced by *ENVISAGE* to the local level using the *SIMPLE-G* model of crop production. Given our focus on nitrogen fertilizer and the MRB, we use the corn–soy version of *SIMPLE-G* (50, 51). *SIMPLE-G-US-CS* is a global partial equilibrium economic model of corn–soy production, consumption and trade, with high spatial resolution within the United States (51). This gridded economic model of US crop production distinguishes more than 48,000 5-arcminute grid cells in which corn and/or soybeans are produced. Each grid cell has the potential for rainfed and irrigated production, and each of these activities exhibits distinct input intensities and yields. Importantly, the land supply elasticities, intensification, and leaching responses vary by location and activity. At each grid cell, crop production and input usage reflect different farming practices (e.g., continuous corn, corn–soy rotation, etc.) and distinguish rainfed and irrigated production activities. The production functions follow a multinesting constant elasticity of substitution (CES) functional form (*SI Appendix*, Fig. S3) with the top-level elasticity of substitution calibrated to reproduce the yield response to the application of nitrogen fertilizer, as obtained from *Agro-IBIS* (52–56). Higher prices for nitrogen fertilizer induce cost-minimizing farmers to reduce application rates, in keeping with grid cell-specific crop fertilization/yield response functions, thereby reducing leaching from the crops’ root zone (Fig. 1). When confronted with higher fertilizer prices, the model predicts farmers’ responses in terms of diminished fertilizer intensity as well as reductions in area planted to corn and soy, which we assumed reverted to crops or natural vegetation according to observed cropping patterns (57) as described in *SI Appendix*. Reductions in corn–soy output result in higher prices and ensuing changes in supply and demand, both within the United States and overseas. Estimated cropping patterns and fertilizer usage are then used to aggregate *Agro-IBIS* estimates of nitrate loss below the root zone, as nitrate is the predominant and most mobile form of nitrogen generated at cropland. Note that our results are likely conservative in understating outcomes because of the focus on direct impacts of increased fertilizer prices, ignoring potential spillovers from the climate policy’s impacts on demand for liquid renewable fuels (e.g., corn ethanol) and livestock.

### *Agro-IBIS*.

Agro-IBIS is a processed-based, rasterized model of agroecology that calculates a suite of agronomic and ecological variables within the soil rooting zone (5, 52, 53). Agro-IBIS was run at a 5-arcminute resolution across the conterminous United States. Spin-up was performed from 1650 to 1947 to generate equilibrium soil biogeochemistry assuming appropriate vegetation cover throughout the epoch and recycling climate inputs from 1948 to 2007. Natural vegetation and a suite of common crops were simulated in all grid cells with agriculture, and natural vegetation was simulated elsewhere. All crops were simulated across a gradient of nitrogen fertilizer application rates (5). Daily predictions of nitrate percolating out of the root zone were aggregated for each landcover prediction provided by *SIMPLE-G-US-CS* and provided as input to *WBM*.

### *WBM*.

The Water Balance Model (*WBM*) (29, 58) is a macroscale, rasterized hydrologic model that incorporates many anthropogenic processes affecting the water cycle, and provides parsimonious representation of the nitrogen cycle in watersheds. The model simulates fluxes of nitrogen as nitrate such as partitioning leachate between runoff through shallow and deep flow paths, accumulation in groundwater, removal in wetlands, and transports nitrate reaching streams through the river network, accounting for in-stream removal, and ultimately estimates nitrate export to the Gulf of Mexico (Fig. 1). Denitrification in wetlands is simulated assuming a well-mixed system with denitrification occurring in the benthic sediments parameterized as a temperature-dependent (*Q*_10_ = 2) process undergoing efficiency loss under increasing concentration. Wetlands intercept nitrate leachate from local croplands underlain by tile drainage prior to entering streams and are assumed to be physically separate from nontile-drained groundwater inputs and upstream riverine flow. *Agro-IBIS* leachate from the root zone provides a boundary condition for simulations spanning 1992 to 2007 at daily time steps. Many landscape features are represented by subpixel processing in the model including impervious and open water area, spatially variable crops, and edge-of-field wetlands.

### Estimating Hypoxia.

The final impacts of nitrogen deliveries to the Gulf hypoxic zone are estimated using an existing model that predicts midsummer peak hypoxic area and volume as a function of average daily loading of total nitrogen during the month of May (7); the response curve for hypoxic area has been updated with data through 2020 from the US Geological Survey. We report the average impacts on hypoxic area and volume based on *Agro-IBIS/WBM* nitrate export to the Gulf of Mexico with perturbations described in the Experimental Design section. Reductions in Mississippi River nitrate loading under various policy and uncertainty scenarios, rescaled to account for other components of total nitrogen and deliveries through the Atchafalaya River mouth (*SI Appendix*, section S.7), are thus translated into reductions in hypoxic area and volume.

### Experimental Design.

We present environmental and economic outputs associated with four assumed carbon prices ($0, $51, $76, and $152/ton CO_2_-e) and three levels (0, 50, and 100 percent) of potential basin-wide crop area that could be restored for treatment by wetlands. Potential areas for restoration were defined by soil properties and subsurface drainage status of cropland (*SI Appendix*, section S.4.3). Restoration ensured that wetlands occupied 0.5% of their catchments, with the remainder of the catchments consisting of solely tile-drained corn–soy crops to maximize nitrate treatment. Generally, the wetland systems we consider are engineered systems treating tile-drainage effluent consistent with the Farmable Wetlands Program (Conservation Reserve Program 2015). Due to computational limitations, the high volume of model variables, and the deep uncertainty associated with the true underlying value of many system parameters, we do not present an exhaustive uncertainty analysis for the model parameters. Instead, we have run an exploratory set of scenarios combining these carbon prices and wetland restoration levels with varying values of three major uncertainties. One is economic, the elasticity of substitution between nitrogen fertilizer and other crop inputs (e.g., land and water). The second is a groundwater storage parameter that transfers a fraction of shallow groundwater to long-term groundwater storage, and the third is the denitrification efficiency of wetlands. Table 1 summarizes the 174-scenario suite of simulations, with additional detail on the parameter values in *SI Appendix*. *SI Appendix*, Table S3 summarizes model assumptions both represented and unrepresented in our model sensitivity analyses and how model results would or would likely be affected by uncertainty in their underlying representation. Results are primarily reported as percentage changes in outcome metrics relative to the null case with no carbon price and no wetland restoration in place, with uncertain parameters taking the baseline values noted in Table 1.

## Supplementary Material

Appendix 01 (PDF)

## Data Availability

Model results data have been deposited in MyGeoHub (59).
