# Supplementary material for: US climate policy yields water quality cobenefits in the Mississippi Basin and Gulf of Mexico
Source: Proc Natl Acad Sci U S A. 2023 Oct 16;120(43):e2302087120. doi: 10.1073/pnas.2302087120 (PMC10614783; doi:10.1073/pnas.2302087120)
Supplement: Supplementary file 1 — Appendix 01 (PDF) [file pnas.2302087120.sapp.pdf]

PNAS

Supporting Information for

US climate policy yields water quality co-benefits in the Mississippi Basin and Gulf of Mexico

Shan Zuidema, Jing Liu, Maksym G. Chepeliev, David R. Johnson, Uris L. Baldos, Stephen Frolking, Christopher J. Kucharik, Wilfred M. Wollheim, and Thomas W. Hertel

Shan Zuidema
Email: shan.zuidema@unh.edu

This PDF file includes:

Supporting Text

S.1 State and local climate policy initiatives 2
S.2 ENVISAGE computable general equilibrium model 2
S.3.1 Carbon pricing scenario framework 7
S.3.2 Timeframe of the analysis and the cost of ammonia production 9
S.3.3 Ammonia production using electrolysis and the impact of carbon pricing 10
S.4 The Agro-IBIS model 11
S.5 The SIMPLE-G-US-CS model 13
S.5.1 Main structure of the model 13
S.5.2 Build biophysical relationship into the economic model 14
S.5.3 Identify wetland extent in SIMPLE-G-US-CS model 16
S.5.3 Aggregating crop area and fertilization rates 16
S.6 Simulating watershed fate of nitrate with WBM 19
S.7 Modeling hypoxic area and volume 19
S.8 Inter-model sensitivities 20
SI References 23

Figures

Figure S 1. Carbon pricing scenario framework. 7
Figure S 2. Selected carbon pricing impacts estimated by ENVISAGE model 8
Figure S 3. Conceptual diagram illustrating the output structure of the SIMPLE-G-US-CS 15
Figure S 4. Yield response and annual nitrate leaching response. 15

Tables

Table S 1. Regional concordance for ENVISAGE model 3
Table S 2. Activities' concordance for ENVISAGE model 4
Table S.3. Comparison of the ammonia production cost structures reported in selected studies…10
Table S 4: Baseline and changes to land allocation 17
Table S 5: Baseline and changes to Agro-IBIS nitrate leachate.... 18
Table S6: Core assumptions, inputs, and parameters used 21

S.1 State and local climate policy initiatives

Until recently, most of the climate policy initiatives in the US have been undertaken at the state and local level. California has an emission trading scheme (ETS) with the Canadian province of Québec that covers many large emitters. The stated goal of California is to require emissions to fall 40% from 1990 levels by 2030. Critics of the program are worried that it is failing to achieve its objectives as in 2019 the state's emissions were only 3% below 1990 level (1). Allowance prices have ranged from $10-20 per tonne of CO2 from 2015 through late 2021, but have recently jumped to over $30 (2). The other major ETS initiative in the U.S. involves the Regional Greenhouse Gas Initiative (RGGI). RGGI puts a cap on emissions from power plants (with a capacity over 25 megawatts) in 11 northeastern states. The current objective is to lower emissions by 30% between 2020 and 2030 (RGGI 2017). Prices since 2010 have been very modest, mostly in the range of $2-6 per tonne of CO2, but have moved up sharply since the end of 2020 to reach around $14 in 2022 (RGGI 2022). Beyond ETS regimes, many states have implemented a variety of other policies to reduce greenhouse gas emissions, for example, 30 states have renewable portfolio standards in the power sector (4), and also have various measures and incentives to improve energy efficiency and switch to cleaner energy sources.

S.2 ENVISAGE computable general equilibrium model

A static computable general equilibrium (CGE) model is used to quantify the economic impacts of climate mitigation policies (5). The model follows the circular flow of an economy paradigm. Firms purchase input factors (for example labor and capital) to produce goods and services. Households receive the factor income and in turn demand the goods and services produced by firms. Equality of supply and demand determines equilibrium prices for factors, goods and services. Production is implemented as a series of nested constant-elasticity-of-substitution (CES) functions the aim of which is to capture the substitutability across all inputs.

Income accrues from payments to factors of production and is allocated to households (after taxes). The government sector accrues all net tax payments and purchases goods and services. The model incorporates multiple utility functions for determining household demand—for this paper, the constant-differences-in-elasticities (CDE) utility function was chosen. Trade is modeled using the so-called Armington specification that posits that demand for goods is differentiated by region of origin (5). The model allows for domestic/import sourcing at the aggregate level (after aggregating domestic absorption across all agents), or at the agent-level.

The model has two fundamental markets for goods and services. Domestically produced goods sold on the domestic market, and domestically produced goods sold by region of destination. All other goods and services are composite bundles of these goods. Two market equilibrium conditions are needed to clear these two markets.

The model applied in the study incorporates three types of production factors: 1) labor (disaggregated into skilled and unskilled); 2) capital; and 3) land. An assumption of full employment was used for the simulations. Capital is allocated across sectors so as to equalize rates of returns.

ENVISAGE incorporates the main greenhouse gases—carbon dioxide (CO_2), methane, nitrous oxide, and fluorinated gases, though in the current study, for the carbon pricing coverage, we focus only on CO_2 emissions from fossil fuel combustion. Reported changes in the CO2 emissions include both fossil fuel combustion CO2, as well as CO2 from industrial processes. A number of carbon control regimes are available in the model. The incidence of the carbon tax allows for partial or full exemption by commodity and end-user. The model allows for emission caps in a flexible manner—where regions/sectors can be segmented into coalitions.

Key data input that underlies the analysis with ENVISAGE model is the Global Trade Analysis Project (GTAP) Power database version 10(6). The GTAP-Power 10 database represents a snapshot of the global economy for 2014 reference year, covering 141 regions and 76 activities, including a detailed representation of electricity and heat generation.

When the global database like GTAP is used for modelling applications, a conventional practice is to design a specific sectoral and regional aggregation that would result in a feasible (from the computational point of view) model size and would simultaneously provide enough level of details for the sectors and regions of interest. In this context, when designing sectoral aggregation, we focused on keeping a high level of details in primary and processed agricultural sectors (including corn and soybeans) and food processing activities, as well as energy sector (fossil fuels and electricity generation activities). In part of corn and soybean representation, we are using the most-detailed data available in the GTAP Data Base. Corn and soybean are represented within two separate sectors - “Other grains” and “Oil seeds” respectively.

On the regional aggregation side, we represent US as a single country and cover key US trading partners and large economies as individual countries/regions, including EU, China, Brazil, India, Japan and Russia. The rest of the world is aggregated into seven composite regions based on the geographical basis (Table S 1). Earlier studies that show that carbon prices and economy-wide leakage rates are mostly unaffected by the degree of sectoral aggregation within a CGE model application (7).

Another important input to the parametrization of the ENVISAGE CGE model is a set of substitution and transformation elasticities. These parameters define the behavioral reactions of agents (producers, consumers, government) to the shocks implemented within the modelling framework. In the case of ENVISAGE model, most of the substitution, transformation and consumer demand elasticities are sourced from the GTAP database (8). The latter relies on a number of econometric estimates and other data sources to derive the values of corresponding parameters.

Table S 1. Regional concordance for ENVISAGE model

No.	Region	GTAP Concordance
1	United States of America (USA)	United States of America (USA)
2	China (CHN)	China (CHN)
3	India (IND)	India (IND)
4	Japan (JPN)	Japan (JPN)
5	Brazil (BRA)	Brazil (BRA)
6	Russia (RUS)	Russian Federation (RUS)
7	Rest of East Asia (XEA)	Rest of Oceania (XOC), Mongolia (MNG), Rest of East Asia (XEA), Brunei Darussalam (BRN), Cambodia (KHM), Indonesia (IDN), Laos (LAO), Malaysia (MYS), Philippines (PHL), Thailand (THA), Viet Nam (VNM), Rest of Southeast Asia (XSE)
8	Rest of South Asia (XAS)	Bangladesh (BGD), Nepal (NPL), Pakistan (PAK), Sri Lanka (LKA), Rest of South Asia (XSA)

No.	Region	GTAP Concordance
**9**	Rest of Europe & Central Asia (XEC)	Albania (ALB), Belarus (BLR), Ukraine (UKR), Rest of Eastern Europe (XEE), Rest of Europe (XER), Kazakhstan (KAZ), Kyrgyzstan (KGZ), Tajikistan (TJK), Rest of Former Soviet Union (XSU), Armenia (ARM), Azerbaijan (AZE), Georgia (GEO), Turkey (TUR)
**10**	Middle East & North Africa (MNA)	Bahrain (BHR), Iran (IRN), Israel (ISR), Jordan (JOR), Kuwait (KWT), Oman (OMN), Qatar (QAT), Saudi Arabia (SAU), United Arab Emirates (ARE), Rest of Western Asia (XWS), Egypt (EGY), Morocco (MAR), Tunisia (TUN), Rest of North Africa (XNF)
**11**	Sub-Saharan Africa (SSA)	Benin (BEN), Burkina Faso (BFA), Cameroon (CMR), Côte d'Ivoire (CIV), Ghana (GHA), Guinea (GIN), Nigeria (NGA), Senegal (SEN), Togo (TGO), Rest of Western Africa (XWF), Central Africa (XCF), South-Central Africa (XAC), Ethiopia (ETH), Kenya (KEN), Madagascar (MDG), Malawi (MWI), Mauritius (MUS), Mozambique (MOZ), Rwanda (RWA), Tanzania (TZA), Uganda (UGA), Zambia (ZMB), Zimbabwe (ZWE), Rest of Eastern Africa (XEC), Botswana (BWA), Namibia (NAM), South Africa (ZAF), Rest of South African Customs Union (XSC)
**12**	Rest of Latin America & Caribbean (XLC)	Mexico (MEX), Argentina (ARG), Bolivia (BOL), Chile (CHL), Colombia (COL), Ecuador (ECU), Paraguay (PRY), Peru (PER), Uruguay (URY), Venezuela (VEN), Rest of South America (XSM), Costa Rica (CRI), Guatemala (GTM), Honduras (HND), Nicaragua (NIC), Panama (PAN), El Salvador (SLV), Rest of Central America (XCA), Dominican Republic (DOM), Jamaica (JAM), Puerto Rico (PRI), Trinidad and Tobago (TTO), Rest of Caribbean (XCB)
**13**	European Union & other Western Europe (WEU)	Rest of North America (XNA), Austria (AUT), Belgium (BEL), Cyprus (CYP), Czech Republic (CZE), Denmark (DNK), Estonia (EST), Finland (FIN), France (FRA), Germany (DEU), Greece (GRC), Hungary (HUN), Ireland (IRL), Italy (ITA), Latvia (LVA), Lithuania (LTU), Luxembourg (LUX), Malta (MLT), Netherlands (NLD), Poland (POL), Portugal (PRT), Slovakia (SVK), Slovenia (SVN), Spain (ESP), Sweden (SWE), United Kingdom (GBR), Switzerland (CHE), Norway (NOR), Rest of EFTA (XEF), Bulgaria (BGR), Croatia (HRV), Romania (ROU), Rest of the World (XTW)
**14**	Other high-income (XHY)	Australia (AUS), New Zealand (NZL), Hong Kong (HKG), Korea (KOR), Taiwan (TWN), Singapore (SGP), Canada (CAN)

Table S 2. Activities' concordance for ENVISAGE model

No.	Activity	GTAP Concordance
**1**	Rice (RIC)	Paddy rice (PDR), Processed rice (PCR)
**2**	Wheat (WHT)	Wheat (WHT)
**3**	Other grains (GRO)	Cereal grains nec (GRO)

No.	Activity	GTAP Concordance
4	Vegetables and fruits (V_F)	Vegetables, fruit, nuts (V_F)
5	Oil seeds (OSD)	Oil seeds (OSD)
6	Sugar (SUG)	Sugar cane, sugar beet (C_B), Sugar (SGR)
7	Other crops (OCR)	Plant-based fibers (PFB), Crops nec (OCR)
8	Cattle, etc. (CTL)	Bovine cattle, sheep and goats, horses (CTL), Wool, silk-worm cocoons (WOL)
9	Other livestock (OAP)	Animal products nec (OAP)
10	Raw milk (RMK)	Raw milk (RMK)
11	Forestry (FRS)	Forestry (FRS)
12	Coal extraction (COA)	Coal (COA)
13	Oil extraction (OIL)	Oil (OIL)
14	Gas extraction and distribution (GAS)	Gas (GAS), Gas manufacture, distribution (GDT)
15	Other extraction (OXT)	Other Extraction (formerly omn Minerals nec) (OXT)
16	Bovine meat products (CMT)	Bovine meat products (CMT)
17	Other meat products (OMT)	Meat products nec (OMT)
18	Dairy products (MIL)	Dairy products (MIL)
19	Other processed foods (OFD)	Fishing (FSH), Vegetable oils and fats (VOL), Food products nec (OFD)
20	Beverages & tobacco (B_T)	Beverages and tobacco products (B_T)
21	Energy intensive manufacturing (KE5)	Paper products, publishing (PPP), Rubber and plastic products (RPP), Mineral products nec (NMM), Ferrous metals (I_S), Metals nec (NFM)
22	Refined oil products (P_C)	Petroleum, coal products (P_C)
23	Chemical products (CHM)	Chemical products (CHM)
24	Equipment manufacturing (MEQ)	Computer, electronic and optical products (ELE), Electrical equipment (EEQ), Machinery and equipment nec (OME), Motor vehicles and parts (MVH), Transport equipment nec (OTN)

No.	Activity	GTAP Concordance
**25**	Other manufacturing (XMN)	Textiles (TEX), Wearing apparel (WAP), Leather products (LEA), Wood products (LUM), Basic pharmaceutical products (BPH), Metal products (FMP), Manufactures nec (OMF)
**26**	Electricity transmission & distribution (ETD)	Electricity transmission and distribution (TnD)
**27**	Coal-powered electricity (ELC)	Coal power baseload (CoalBL)
**28**	Oil-powered electricity (ELO)	Oil power baseload (OilBL), Oil power peakload (OilP)
**29**	Gas-powered electricity (ELG)	Gas power baseload (GasBL), Gas power peakload (GasP)
**30**	Nuclear-powered electricity (ELN)	Nuclear power (NuclearBL)
**31**	Hydro-electricity (ELH)	Hydro power baseload (HydroBL), Hydro power peakload (HydroP)
**32**	Wind-powered electricity (ELW)	Wind power (WindBL)
**33**	Solar-powered electricity (ELS)	Solar power (SolarP)
**34**	Other renewable electricity (ELX)	Other baseload (OtherBL)
**35**	Construction (CNS)	Construction (CNS)
**36**	Trade services (TRD)	Trade (TRD), Warehousing and support activities (WHS)
**37**	Terrestrial transport (OTP)	Transport nec (OTP)
**38**	Water transport (WTP)	Water transport (WTP)
**39**	Air transport (ATP)	Air transport (ATP)
**40**	Private services (PSV)	Accommodation, Food and service activities (AFS), Communication (CMN), Financial services nec (OFI), Insurance (formerly isr) (INS), Real estate activities (RSA), Business services nec (OBS), Recreational and other services (ROS), Dwellings (DWE)
**41**	Government services (GSV)	Water (WTR), Public Administration and defense (OSG), Education (EDU), Human health and social work activities (HHT)

S.3 Carbon pricing and the cost of ammonia production

S.3.1 Carbon pricing scenario framework

Our carbon pricing scenario framework starts from the reference case represented by the 2014 base year of the GTAP-Power Data Base (6) (Figure S.1). We first implement the carbon price in the European Union (EU), as observed in the EU's Emission Trading System (ETS) as of April 2022. The level of implemented carbon price is 89 EUR per tCO2-eq (9). Carbon price covers all fossil fuel combustion activities, including households. In addition to economy-wide carbon pricing in the EU, we implement carbon border adjustment (CBA) measures. The CBA mechanism is aimed at protecting domestic industries, avoiding carbon leakage and preventing the importation of additional carbon intensive products from sources with less stringent environmental regulations than in the EU. The CBA mechanism is implemented in a form of levy on the carbon content of imported commodities that are entering the EU. The levy is defined based on the difference in carbon prices applied by the EU and the source of commodity imports following an approach outlined in (10). Within such implementation we consider direct emissions from fuel combustion only (Scope 1), indirect emissions from heat and electricity use (Scope 2), as well as emissions embodied into other intermediate inputs within the production process (i.e. Scope 3).

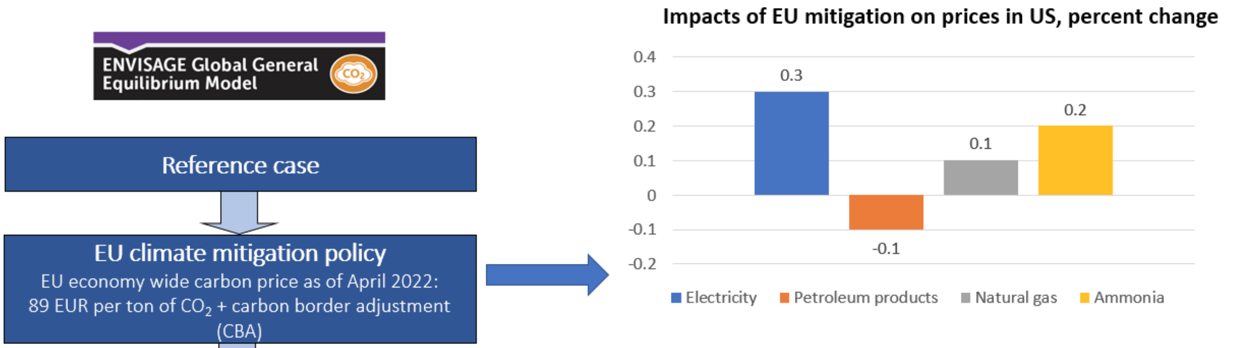

Social Cost of CO_2, 2020 – 2050 (in 2020 dollars per metric ton of CO_2)

	Discount Rate and Statistic			
Emissions Year	5% Average	3% Average	2.5% Average	3% 95th Percentile
2020	14	51	76	152
2025	17	56	83	169
2030	19	62	89	187
2035	22	67	96	206
2040	25	73	103	225
2045	28	79	110	242
2050	32	85	116	260

Figure S 1. Carbon pricing scenario framework.

Implementation of the EU economy-wide carbon pricing and CBA measures has a moderate impact on the US economy, as changes in energy and fertilizer prices vary in a range of 0.1%-0.3% (Figure 1). After implementing carbon price and CBA measures in the EU, we construct a new reference, which we use as a starting point for the assessment of the US climate policy impacts. For the case of the US, we consider three mitigation scenarios – each involving a different social cost of carbon: $US 51, 76 and 152 per tCO2e, reflecting the underlying uncertainty in the appropriate social discount rate as well as in the science of climate impacts (11). Implemented in the US carbon prices are further complemented by the CBA measures, similar to the case of the EU discussed above. Incorporation of the CBA into designed modeling framework allows us to capture a more realistic

potential set up of the mitigation policies. Without a CBA implementation, a likely outcome of the carbon pricing in US would be a substitution of domestic fertilizer production by imported fertilizer(12), which would largely reduce potential co-benefits of mitigation policies in terms of nitrogen leaching reduction, as well as lead to larger burden on domestic fertilizer producers, as they would be losing competitiveness relative to the foreign fertilizer producers in countries with less stringent mitigation policies.

When implementing carbon pricing in the US, we fix the domestic producer prices of corn and soy (at the reference year level) by adjusting the production subsidy level in the ENVISAGE model. With such approach, we facilitate a more refined model linkage between ENVISAGE and SIMPLE-G, allowing each model to capture a channel of impacts that it is most appropriate for. The partial equilibrium SIMPLE-G model, which has a detailed representation of corn-soy production activities, captures the supply-side effects of carbon pricing within US agriculture, while an economy-wide ENVISAGE model represents the demand-side impacts.

Implemented carbon pricing policies lead to substantial increases in US energy prices (Figure S 2a). In the case of natural gas, the carbon tax-inclusive price increases in a range of 58.8%-176.2% depending on the scenario. Since natural gas is a key input to ammonia production (Figure S 2b), prices of ammonia increase by 30.4-90.6% across scenarios (Figure S 2c).

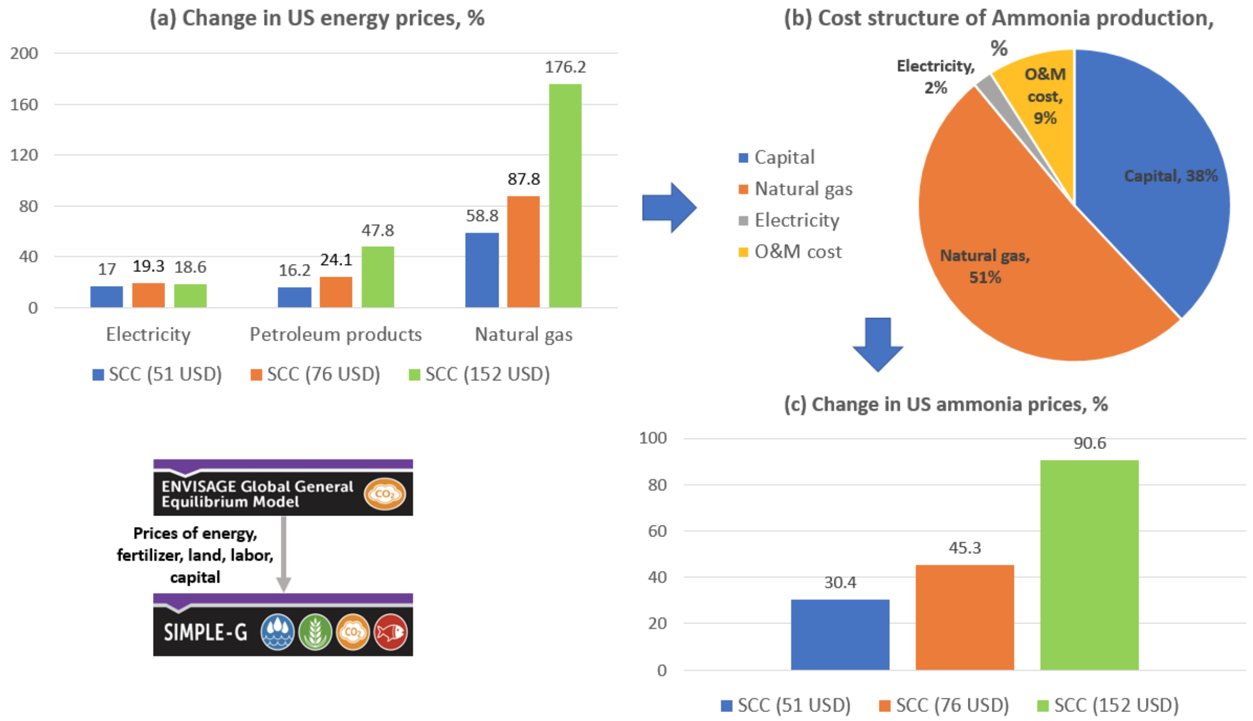

Figure S 2. Selected carbon pricing impacts estimated by ENVISAGE model

The GTAP-Power 10 database, being the most up-to-date publicly available version of the GTAP-Power database, represents a global economy for the historical (2014) reference year, while carbon pricing scenarios considered in the study correspond to the forward-looking policies. This naturally creates a gap between the economic structures represented in the developed modelling framework and the state of the economy, which would face the carbon pricing in the future. A direct implication is that the conducted assessment of carbon policies in US should be interpreted as an upper bound of the corresponding mitigation impacts (in terms of emission reductions relative to the reference case). With improvements in energy efficiency, increasing share of clean energy sources and

declining share of energy-intensive manufacturing sectors over time, economy-wide emissions tend to decline (13, 14). Between 2014 and 2021 CO_2 emission intensity of the world GDP has decreased by 13 percent, while that of US by 22 percent (15). These trends are expected to continue. As a result, application of the same level of carbon price over an economy with lower emission intensity (than the reference case) would result in a less significant increase in prices of the energy-intensive goods and services and overall lower impact on emission reductions. At the same time, energy and emission intensity of various production and consumption processes changes differently over time. While US emission intensity of electricity and transportation activities could be expected to experience a relatively substantial decline over time (due to increasing share of clean generation technologies and rising electrification rates in transportation), such production processes like ammonia fertilizer have relatively limited scope for energy and emission intensity improvements. In many cases, fertilizer emission factors estimated by the Intergovernmental Panel on Climate Change 2006 report are used for calculating and projecting emissions from the fertilizer production (16), indicating a relatively high stability of the corresponding production processes. Since fertilizer emission intensity is the key driver of the carbon pricing implications within the designed model linkages (between ENVISAGE and SIMPLE-G), there is a relatively low uncertainty associated with a temporal evolution of the corresponding production process.

S.3.2 Timeframe of the analysis and the cost of ammonia production

In the context of alternative ammonia production technologies, it should be noted that our analysis focuses on the medium-term climate mitigation perspective considering that all models involved in the assessment are static and do not capture the long-term trends of the evolution in economic, energy and agricultural systems. While ammonia production through electrolysis that relies on renewable electricity is a promising production pathway, as of today there are no commercial large-scale plants that produce ammonia using this technology (17, 18). As recognized in the recent report by the International Energy Agency (19), “challenges remain in the use of hydrogen produced from variable renewable energy (such as solar PV and wind) directly in captive installation arrangements.” The same report estimates that even under the most ambitious net-zero scenario, in 2030, the global ammonia production process would be primarily relying on natural gas as a feedstock with a share of electrolysis representing less than 7% of global ammonia production (19). The commercial feasibility of the electrolysis production process would substantially increase post-2030. The most recent release of the Energy Technology Perspectives (20) estimates that as of 2022, the average cost of producing ammonia in the United States using a combination of electrolysis and solar photovoltaics (PV) is almost 3 times higher compared to the steam methane reforming production pathway.

The implementation of carbon prices in the medium term does not necessarily mean rapid/immediate decarbonization of hard-to-abate sectors, such as the ammonia production process. In this regard, our scenario set up for the case of the U.S. closely follows mitigation efforts currently implemented in the EU, where selected sectors, including fertilizer production, are facing a carbon price in the range of 80-100 EUR per tonne of CO2-equivalents (9), while the ammonia production process still largely relies on the fossil fuel feedstocks and is expected to remain so during the coming years. Though even assuming that the electrolysis production technology would become available at a substantial scale by the end of this decade, the price of the ammonia produced using this technology, under competitive market assumptions, would be largely driven by the price of the ammonia produced using the conventional (fossil fuel-based) manufacturing process, and thus the fertilizer price increases faced by consumers are expected to be closely aligned to those estimated in the paper for the case of conventional ammonia production technology. The corresponding market mechanism might not be in action in the long run (e.g. post-2030), when the volumes of ammonia production using electrolysis would dominate the market, but this timeframe goes beyond the scope of the current assessment.

Noting this, we recognize that it is important to better explain and justify the cost assumptions behind the pricing of the ammonia fertilizer used in this paper. For the conducted assessment we assumed that the ammonia cost structure follows (21) for the case of a medium ammonia plant size with an output of 516 Kt per year. This capacity is within range of a typical ammonia plant size, which ranges between 200 Kt and 1200 Kt per year(19). Depending on the technological assumptions, an alternative cost structure might be used to represent the ammonia production process. Table S.3 provides a comparison of the ammonia cost structure applied in the current study and the cost structures reported in the available literature. The share of natural gas ranges between 49% (20) and 56%(22), which is consistent with a 51% cost share used in the current study.

Table S.3. Comparison of the ammonia production cost structures reported in selected studies.

**Data source**	IEA (2023a)	Maung et al. (2012) – ***used in this study***	Cloete et al. (2021)
**Region**	United States	North America (North Dakota)	Europe
**Cost structure, %**			
Capital	34	38	29
Natural gas	49	51	56
Electricity	1	2	0
Operation and maintenance costs/other costs	16	9	15
**Total**	**100**	**100**	**100**

Notes: numbers are rounded to ensure that the total costs sum up to 100%.

S.3.3 Ammonia production using electrolysis and the impact of carbon pricing

Based on the IEA (23) estimates, the indicative production costs for ammonia via electrolysis in the United States are expected to decline substantially by 2030. While average 2022 ammonia production costs via electrolysis and variable renewable energy in the U.S. are in a range of 893-1410 USD/tonne (compared to around 400 USD/tonne for the fossil-based production process), by 2030 corresponding costs are expected to be in a range of 561-787 USD/tonne or 40%-97% higher than the current production costs using fossil feedstocks (23). In this regard, it is reasonable to expect that by 2030 under the assumption of high carbon prices, where the implied cost of ammonia production increases beyond the expected cost of the electrolysis production process, the latter would become economically feasible and competitive with the conventional (fossil-based) ammonia. The lower range of the green ammonia production costs broadly corresponds to the ammonia price increases observed under the SCC of $76 (+46.6%), while the upper bound of the green ammonia manufacturing costs is well aligned with the ammonia price increases under the SCC of $152 (+93.3%). In this regard, our results, when combined with the projected costs of green ammonia production in 2030, imply that under an optimistic electrolysis cost scenario, any mitigation efforts with SCC beyond $76 would be equivalent to the impacts observed under the SCC $76 scenario. Under the pessimistic assumption regarding the evolution of green ammonia production costs, the electrolysis technology would become economically feasible in 2030 only under the SCC above $152.

S.4 The Agro-IBIS model

S.4.1. Model description

An agroecosystem model, Agro-IBIS (24, 25) was used in simulations under various weather conditions and fertilizer application rates across the study region. Agro-IBIS is a processed-based, rasterized model of agroecology that calculates a suite of agronomic and ecological variables within the soil rooting zone. Agro-IBIS was run at a 5-arcminute resolution across the conterminous United States. Agro-IBIS simulates both biogeochemical and biophysical processes and accounts for the exchange of water, carbon, energy, and nitrogen between the soil, plant, and atmosphere. Leaf level interactions, including photosynthesis and stomatal conductance, are initially calculated at an hourly temporal resolution (27, 28). These variables are then scaled up to the canopy level and updated at the daily to yearly temporal resolutions(29, 30). Agro-IBIS calculates water and nutrient balances in the soil-plant-atmosphere system and calculates nitrate leaching below the root zone at 1.5m. Agro-IBIS assumes all nitrate leaves the root zone via infiltration below the soil with no direct surface runoff. In this study, Agro-IBIS output is pixel specific fluxes for each of 8 land-cover classes over agricultural areas of the MRB: 1) irrigated maize, 2) rainfed maize, 3) irrigated soy, 4) rainfed soy, 5) irrigated soy/maize rotation, 6) rainfed soy/maize rotation, 7) rainfed wheat, 8) natural vegetation. These fluxes were aggregated based on crop area, crop selection, and fertilization rates determined by SIMPLE-G-US-CS and described below. Agro-IBIS, along with the various inputs required to run this version and resolution, have been documented and validated in previous studies (e.g.(31, 32)). More details are provided below on model input datasets, simulations, assumptions, and evaluation.

S.4.2. Model input datasets and simulation procedure

This study uses all model input data previously described in(33). The Agro-IBIS model was driven by the ZedX Inc. (Bellefonte, PA) observation based daily weather dataset (temperature, precipitation, humidity, solar radiation, and wind speed) which is at the 5 arcmin spatial resolution for the entire CONUS. Soil texture data was obtained from USDA State Soil Geographic Database (STATSGO) (34) and previously described in(31). Crops received nitrogen fertilizer and manure inputs at “baseline” rates consistent with previous data (26) for the period 1950 through 1989 and then EarthStat fertilizer and manure rates were used from 1990 to 2007 (available at http://www.earthstat.org/). NASA GISS estimates of changing atmospheric CO_2 from 1650-2007 were used to parameterize Agro-IBIS simulations. During the simulations that approximated contemporary conditions, atmospheric CO_2 concentration was held fixed at 391 ppm.

All model scenarios are a “restart run” of 60 years in length using a transient time series of climate and weather for the 1948-2007 time period based on the daily gridded ZedX (5min by 5min) climate dataset. Simulations build upon a spin-up simulation that spanned 358 years (1650 to 2007), which was necessary to bring soil biogeochemistry (e.g., coupled C and N cycling) to an equilibrium state. During spin up years from 1650-1947, the 60-year ZedX daily climate dataset was “recycled” so that the year 1650 was actual weather year 1948, sequentially stepping through all years in sequence, and then restarting from the beginning when reaching the end of the time series. While the last 60 years of the restart runs were 2008-2067 in model years, they actually denote climate years of 1948-2007. The first 10 years of the output data was discarded due to a new equilibrium needing to be reached after restart runs commence. Thus, the last 50 years of each simulation can be used and denotes the actual timeseries of climate/weather from 1958-2007.

S.4.3. Model assumptions

The following logic and assumptions were used when applying N to cropping systems:

1. For continuous maize, N from mineral fertilizer and manure was applied at the planting date which is automatically determined by the model based on seasonal weather.

2. For maize/soybean rotations, N fertilizer connected to soybeans in the EarthStat.org datasets were applied after fall harvest of soybeans, with N for maize applied at springtime planting date.

3. For continuous winter wheat, 33% of N was applied at time of fall planting, with 67% of N applied on the following January 1.

4. For scenarios that represented varied N applications with irrigation, a threshold of 50% plant available water [(actual VWC – PWP VWC)/(FC VWC – PWP VWC)] in the top 60cm of soil was used as the irrigation trigger in every grid cell. VWC denotes "volumetric water content"; PWP = permanent wilting point; FC = Field capacity. Irrigation was applied during a nominal 6-hour event to a grid cell and assumed that a maximum daily amount applied is 50mm. Irrigation was only applied to corn phase of rotations and was only turned on when corn was actively growing.
The following suite of scenario simulations were performed with Agro-IBIS to generate yield and nitrate leaching response curves for cropping systems and nitrate leaching from other systems:

- Irrigated maize with varied levels of N fertilizer and manure applied (0, 20%, 60%, 80%, 90%, 100% of baseline)
- Rainfed maize with varied levels of N fertilizer and manure applied (0, 20%, 60%, 80%, 90%, 100%of baseline)
- Irrigation and rainfed soy
- Irrigated maize/soybean rotation, with varied levels of N fertilizer and manure applied to maize (0, 20%, 60%, 80%, 90%, 100%of baseline)
- Rainfed maize/soybean rotation, with varied levels of N fertilizer and manure applied to maize (0, 20%, 60%, 80%, 90%, 100%of baseline)
- Rainfed continuous winter wheat with varied levels of N fertilizer and manure applied (0, 20%, 60%, 80%, 90%, 100%of baseline)
- Natural vegetation (grasses/trees) with no additional N inputs other than atmospheric deposition

S.4.4. Model evaluation

The version of Agro-IBIS used in this study has been previously parameterized, calibrated and evaluated using both site level data as well as USDA county level crop yield data across the Mississippi Basin for both rainfed and irrigated crops (e.g.(24, 25)). Yield is only one variable that has been used in the continuous model improvement and evaluation process given the close connections between carbon, water, energy, and nutrient cycling in a process-based model like Agro-IBIS. Multiple aspects of model performance have been assessed to ensure that simulated output variables, and in particular crop yield and nitrate leaching, are correct for the right reasons. Therefore, we have utilized field experiments and AmeriFlux data (e.g. (35–38)) in addition to USDA county level yield data to evaluate model simulated processes (e.g. N cycling and leaching, carbon cycling, water balance) in previous publications (26, 32, 39–44).

S.5 The SIMPLE-G-US-CS model

S.5.1 Main structure of the model

SIMPLE-G-US-CS is a global partial equilibrium economic model with spatial details describing crop productions and agricultural inputs use. The equilibrium for sluggish and semi-mobile inputs (e.g., cropland and water) is established at the grid-cell level, while the markets for mobile inputs (e.g., fertilizer, labor, and capital) are cleared at the regional level. We chose a partial equilibrium model to focus on specific market in isolation, given that the central interest of present study is to estimate the leaching response to carbon policy and compare it with other conservation practice, specifically wetland that is identified as one of the most effective leaching removal practices. Our analysis does not mean to examine the overall welfare effect resulted from carbon policy. That will require a general equilibrium model that captures the feedback between markets, which is beyond the scope of the study.

SIMPLE-G-US-CS is a specialized version of the SIMPLE-G-US model (45) focusing on two crops: corn and soybeans. More descriptions of the SIMPLE-G-US model can be found in (45, 46). Because the prices for these two crops tend to move in tandem (they are close substitutes, both in production and in use), they can be combined into a single composite crop for purposes of capturing long-run equilibrium effect, but not the short-term market volatility. The rising Nitrogen fertilizer cost caused by carbon tax may initially sway farmers toward soybeans. However, continuous increase in soybean supply will eventually drive the price of soybeans down. Similarly, the migration from corn to soybeans will reduce corn supply, driving the price of corn up. The adjustment made by the market will try to restore an historic price ratio between the two crops. The crop aggregation avoids the complexity arising from the cross elasticities of demand and supply (i.e., the relationship between the two products when the price of one of them changes), which is not the focus of the model. The corn-soy composite is obtained by converting soybean production into (price-weighted) corn-equivalent tons. At each grid cell, crop production and input usage reflect different types of production (e.g., continuous corn, corn-soy rotation, etc.).

The production functions follow a multi-nesting constant elasticity of substitution (CES) functional form illustrated in Figure S6 for one single grid-cell. Each production nest is governed by an elasticity of substitution parameter σ that indicates the easiness of switching between inputs within each layer once the relative prices of the two inputs change. A larger value of σ indicates easier substitution between inputs, and thereby more flexible production technology. When the use of one input is restricted by policy or natural constraints, the other input from the same layer will be employed more intensively, with the magnitude of this response governed by the size of the substitution elasticity σ and the extent of the change in relative input scarcity (relative prices). This elasticity was uniquely estimated for the SIMPLE-G-CS model using the crop yield response to Nitrogen fertilizer use provided by the Agro-IBIS model, which will be explained in detail in the subsection S.5.2. The production function—including nitrogen fertilizer intensity, input cost shares, and substitutability of inputs—is unique for each grid cell. The nesting structure differs at the bottom layer between irrigated and rainfed production. For irrigated crops, irrigation water is combined with irrigable land to produce a land-water composite, which is further combined with non-land inputs (e.g., capital and labor) to produce an augmented land input that is finally combined with N fertilizer to produce the ultimate crop output. Most of the elasticities in the SIMPLE-G-CS model are inherited from the original SIMPLE-G model that was calibrated and validated for publication (Baldos et al., 2020).

S.5.2 Build biophysical relationship into the economic model

A defining feature differentiating the corn-soy version of the model from the other SIMPLE-G model variants is nitrogen fertilizer application in crop production and the nitrate leaching module. Given its central importance to the questions addressed in this paper, nitrogen fertilizer application is positioned at the top of the nesting structure, which permits us to pay special attention to the relationship between N and crop production, as well as that between N and leaching. The nitrogen fertilizer application rate data (47) provided the cost share of nitrogen fertilizer at the grid-cell level. The key parameter for this present analysis, the elasticity of substitution between nitrogen fertilizer and the augmented land input (33) which fit transfer functions to the outputs of the Agro-IBIS agroecosystem model (24). Figure S2 shows how this is done for one practice in a single grid cell. By running Agro-IBIS many times (individual dots in Figure S2), we fit a Gompertz curve for each grid cell to the relationship between N applications and crop yield, as well as a quadratic leaching curve characterizing the relationship between N applications under a given practice in a given location and leaching below the root zone. These transfer functions are incorporated into SIMPLE-G-US-CS (33) allowing for a spatially resolved characterization of the tradeoffs between agricultural production and the environment in the SIMPLE-G-US-CS model.

Specifically, the grid-specific Gompertz transfer functions (Equation 1) are used to estimate the elasticity of substitution between Nitrogen fertilizer and the augmented land input following the equation provided by the theory of production economics (Equation 2).

$$f_g = a_g exp^{(-b_g * c_g^n)} \quad \text{(Eq. 1)}$$

where f_g is the normalized production function, n is the fertilizer application rate in kg/ha. Parameters a, b, and c (all index over g for grid-cell) were estimated by fitting the Gompertz function, allowing for a spatially resolved characterization of the tradeoffs between agricultural production and the environment in the SIMPLE-G-US-CS model.

According to Ferguson (48) (page 97, Equation 5.2.18), the elasticity of substitution between N fertilizer and land δ_{LN} can be expressed as a function of f(n) (the Gompertz function itself in Equation 1), f'(n), and f''(n) (the first and second derivative of output with respect to Nitrogen fertilizer application rate).

$$\delta_{LN} = \frac{f_g{}'(n)[f_g(n) - nf_g{}'(n)]}{-nf_g{}''(n)f_g(n)} \quad \text{(Eq. 2)}$$

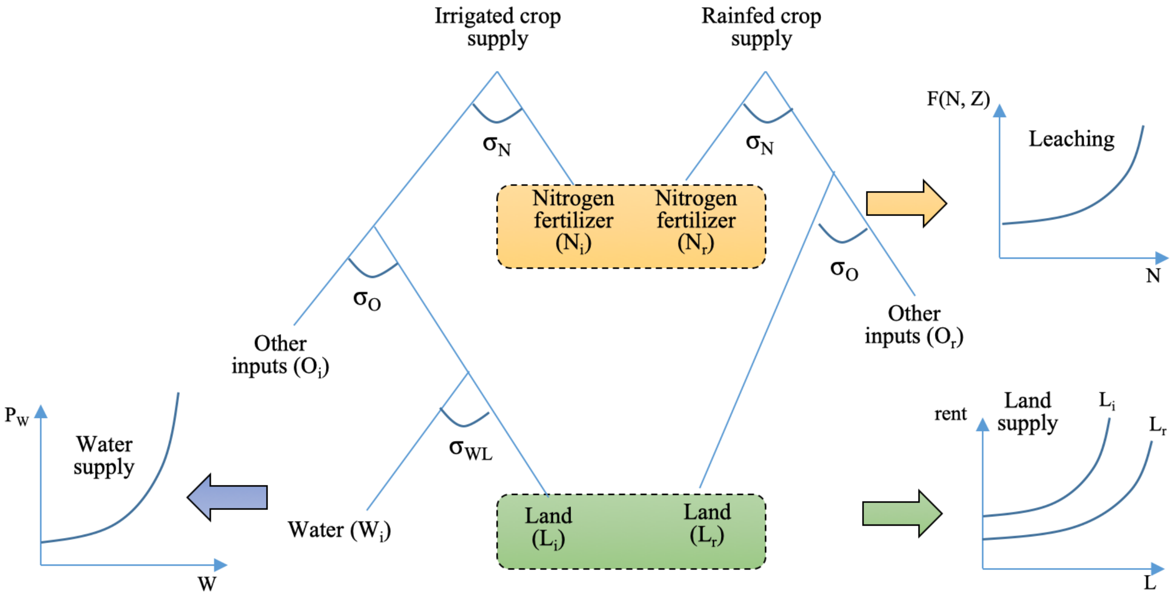

Figure S 3. Conceptual diagram illustrating the output structure of the SIMPLE-G-US-CS model

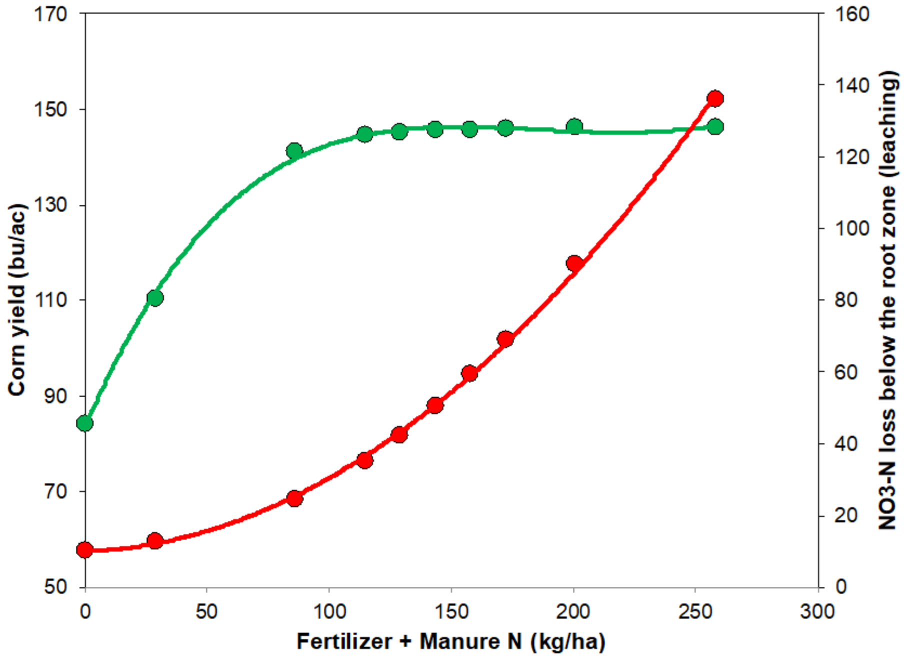

Figure S 4. Yield (bu/ac) response (green) and annual nitrate leaching (kg NO_3-N/ha) response (red) to N fertilizer application rate (kg N/ha).

S.5.3 Identify wetland extent in SIMPLE-G-US-CS model

We assume that all the tile-drained cropland dedicated to corn and soy production will be treated by a wetland encompassing 0.5% areal extent of remaining crops within each grid-cell. This assumption maximizes wetland treatment, by ensuring that wetlands are restored only where catchment areas would be entirely in production. The corn and soybean fields that have installed sub-surface (tile) drainage are targeted, given the evidence that subsurface drains have been found to increase nitrate loss compared with similar undrained soils (49). Then we compare the desirable wetland extent to achieve this maximum treatment capacity with the feasible area of wetland in each grid-cell using maps of hydric soils. An overlapping of the two determines the actual site-specific wetland area to be restored. Following guidelines consistent with prior estimates (50, 51), we assume that a 3.5 acre of grass buffer is required to associate with one acre of wetland basin. That means a total of 4.5 acres is needed to treat a 200-acre tile-drained corn and soybean field. Using this treatment ratio, we calculated the cropland foregone for wetland restoration in each grid-cell.

S.5.3 Aggregating crop area and fertilization rates

The land areas and fertilization rates predicted by SIMPLE-G-US-CS are used to develop rasterized rates of fertilization leachate from the Agro-IBIS model output for introduction to WBM. We define the baseline scenario as a $0 carbon price, no additional wetland restoration, and a mean elasticity of substitution for nitrate fertilizer. For each scenario, the change in land area in corn/soy production relative to the baseline scenario is calculated and crop areas for corn and soy crops are recalculated (Table S4). Then for each scenario, the long-term fraction of fertilizer applied estimated by SIMPLE-G-US-CS relative to the baseline is calculated. This fertilization factor was matched to the nearest fertilization level simulated directly by Agro-IBIS (0%, 20%, 60%, 80%, 90%, or 100%).

For areas where corn/soy lands are removed from production, we assume that croplands would revert to wheat, other crops, or non-crop vegetated lands identified at that location in the MIRCA2000 database (52–54). Where the crop area was identified as corn or soy in MIRCA2000 we assume it reverts to other crops. The other crops category represents all crops other than corn, soy, and wheat and were not explicitly represented by Agro-IBIS simulations for tractability. While a variety of land-uses exist within the basin as represented by the other crops, we assume that all corn and soy croplands that revert to other crops represent undifferentiated grasslands for two reasons:

1) In the early 2000s 77% of new corn/soy cropland expanded from existing grassland (55).
2) Fodder grasses were 4 times more likely to be the second most common crop in pixels where corn or soy were the most common crop in the MIRCA2000 dataset for the Mississippi River Basin.

From the SIMPLE-G-US-CS estimates of cropland area and fertilization factor, we summed nitrogen leachate from the appropriate Agro-IBIS simulation scaled by the relative proportion of each crop at each fertilization level within each pixel. This yielded a weighted average of nitrogen leachate for the crop mix and fertilization levels estimated by SIMPLE-G-US-CS (Table S5). To capture the leaching rate of undifferentiated grasses in our analysis, we attribute the other crops category with a weighted average of leaching rates from Agro-IBIS data for rotated corn/soy (23%) and non-crop vegetation (77%) to approximate the mean leaching rates of perennial crops estimated by (56). The representation of undifferentiated grasses as all other crops not explicitly represented should result in local biases of nitrate leachate where very different crops are grown, for instance rice in Arkansas. These specific crop types were not considered in our analysis.

Table S 4: Baseline and changes to land allocation estimated by SIMPLE-G-US-CS for each scenario considered.

Scenario			Baseline land allocation (and changes) (km^2)			
Carbon price	Fertilizer elasticity	Wetland adoption	Corn/Soy	Wheat	Other	Non-crop
$0	mean	none	**530,443**	**100,631**	**189,397**	**2,065,763**
$0	mean	low	-336	+1.43	+157	+174
$0	mean	high	-687	+2.62	+333	+344
$0	low	low	-313	+1.43	+150	+163
$0	low	high	-678	+2.61	+330	+338
$0	high	low	-326	+1.44	+149	+172
$0	high	high	-705	+2.63	+347	+349
$51	mean	none	-3342	+3.56	+2261	+1105
$51	mean	low	-3661	+4.50	+2415	+1266
$51	mean	high	-3990	+5.34	+2580	+1425
$51	low	none	-3706	+3.93	+2507	+1226
$51	low	low	-4017	+4.81	+2655	+1383
$51	low	high	-4338	+5.64	+2817	+1537
$51	high	none	-3004	+3.19	+2034	+993
$51	high	low	-3331	+4.20	+2192	+1157
$51	high	high	-3665	+5.05	+2359	+1319
$75	mean	none	-5046	+5.05	+3426	+1657
$75	mean	low	-5354	+5.88	+3573	+1811
$75	mean	high	-5668	+6.65	+3732	+1963
$75	low	none	-5594	+5.55	+3797	+1833
$75	low	low	-5892	+6.35	+3941	+1983
$75	low	high	-6197	+7.04	+4095	+2130
$75	high	none	-4535	+4.61	+3077	+1493
$75	high	low	-4856	+5.44	+3233	+1651
$75	high	high	-5180	+6.28	+3397	+1807
$152	mean	none	-10013	+8.86	+6872	+3205
$152	mean	low	-10291	+9.16	+7010	+3342
$152	mean	high	-10576	+9.48	+7158	+3475
$152	low	none	-11123	+9.26	+7654	+3538
$152	low	low	-11388	+9.57	+7785	+3668
$152	low	high	-11660	+9.87	+7927	+3794
$152	high	none	-9001	+8.31	+6163	+2898
$152	high	low	-9291	+8.71	+6308	+3040
$152	high	high	-9588	+9.01	+6461	+3180

Table S 5: Baseline and changes to Agro-IBIS nitrate leachate aggregated from the SIMPLE-G-US-CS land and fertilization conditions in Table S 4.

Scenario			**Nitrate leaching baseline flux** (and changes) ($Gg\ yr^{-1}$)			
Carbon price	Fertilizer elasticity	Wetland adoption	Corn/Soy	Wheat	Other	Non-Crop
$0	mean	none	**1640**	**415**	**187**	**481**
$0	mean	low	-5.78	-0.680	-0.195	+0.004
$0	mean	high	-11.6	-1.35	-0.379	+0.006
$0	low	low	-5.87	-0.690	-0.212	+0.003
$0	low	high	-11.8	-1.37	-0.389	+0.005
$0	high	low	-5.64	-0.671	-0.197	+0.004
$0	high	high	-11.5	-1.33	-0.355	+0.008
$51	mean	none	-77.4	-14.3	-3.90	+0.377
$51	mean	low	-81.5	-14.1	-3.84	+0.378
$51	mean	high	-85.6	-14.0	-3.77	+0.378
$51	low	none	-69.2	-12.8	-2.93	+0.413
$51	low	low	-73.5	-12.7	-2.89	+0.413
$51	low	high	-77.8	-12.6	-2.84	+0.412
$51	high	none	-85.0	-15.7	-4.81	+0.343
$51	high	low	-88.9	-15.5	-4.73	+0.345
$51	high	high	-92.9	-15.3	-4.65	+0.346
$75	mean	none	-110	-20.2	-5.29	+0.556
$75	mean	low	-114	-20.0	-5.23	+0.556
$75	mean	high	-117	-19.9	-5.17	+0.555
$75	low	none	-98.6	-18.1	-3.93	+0.609
$75	low	low	-103	-18.0	-3.89	+0.608
$75	low	high	-107	-17.9	-3.84	+0.606
$75	high	none	-120	-22.1	-6.56	+0.508
$75	high	low	-124	-21.9	-6.48	+0.509
$75	high	high	-127	-21.7	-6.40	+0.508
$152	mean	none	-191	-35.3	-8.59	+1.05
$152	mean	low	-195	-35.1	-8.53	+1.05
$152	mean	high	-198	-34.9	-8.47	+1.04
$152	low	none	-174	-32.0	-6.15	+1.15
$152	low	low	-178	-31.9	-6.11	+1.14
$152	low	high	-182	-31.7	-6.07	+1.14
$152	high	none	-207	-38.4	-10.9	+0.96
$152	high	low	-211	-38.2	-10.8	+0.96
$152	high	high	-214	-38.0	-10.7	+0.95

S.6 Simulating watershed fate of nitrate with WBM

The University of New Hampshire Water Balance Model is a raster based model of macroscale hydrology and biogeochemistry (57–60). Our model of wetland denitrification assumes a well-mixed system with denitrification occurring in the benthic sediments parameterized as a temperature-dependent process (Q_{10}=2), with flow and nitrate mass bypassing wetland processing when wetland water storage exceeds a design maximum depth (61). The overall WBM framework represents the equilibrium fluxes of nitrate through the MRB considering wetlands and historic scenarios of wetland restoration. WBM simulations were performed at a daily time-step. To perform model spinup, WBM was forced with input data from 1996 through 1999 repeated five times, a total of 20 years of model spinup. We then simulated the time period between 1992 and 2007 analyzing output data from all years and summary plots represent mean annual fluxes across those years. WBM was run at a 5-arcminute resolution over a geographic domain covering a domain from 113°55' W to 77°50'W, and 28·55'N to 49°45'N. The evaluated domain covered the Mississippi River drainage basin (MRB) defined by the MERIT 5-arcminute drainage network (62), and following post-processing used to route drainage of small internally draining regions into the larger drainage network.

A fraction (χ_{lost}) of all leachate partitioned to groundwater flowpaths is removed from the surface flow system. The term accounts for long-term net storage term in the vadose zone or deep groundwater and was introduced to reduce a high bias in watershed scale nitrate flux through the river system (61). This parameter was varied to reflect 10% uncertainty in its estimated value, and for each case we calculate the relative difference in deep recharge and export of nitrate from deviations assuming that this property is independent of carbon pricing.

S.7 Modeling hypoxic area and volume

The impacts of nitrogen deliveries on the Gulf hypoxic zone are estimated using the model detailed in (63), which predicts midsummer peak hypoxic area and volume as increasing functions of average daily loading of total nitrogen during the month of May. The response curve for hypoxic volume has been calibrated using nitrogen export data from 1985 to 2011, while the calibration for hypoxic area has been updated with data from 1985 to 2020 from the US Geological Survey; as a result, the model is the same as described in (63), but with updated calibration for hypoxic area provided courtesy of Donald Scavia. We report the average impacts on hypoxic area and volume over this time period based on Agro-IBIS/WBM output covering a consistent 16-year period from 1992 to 2007, calibrated to consistent observations, and perturbed based on the experimental design described above. WBM tracks nitrate loads delivered to the Gulf of Mexico from the Mississippi River; we assume that nitrites, ammonium, and dissolved organic nitrogen are relatively constant (64), with combined loads estimated at 44% of average nitrate loads from 1980 to 2021. Total nitrogen deliveries at the Atchafalaya River mouth are assumed to be 36.2% of those at the mouth of the Mississippi River, an assumption of the model from (63) with updated calibration data. Reductions in Mississippi River nitrate loading under various policy and uncertainty scenarios are therefore translated into reductions in hypoxic area and volume, accounting for these additional components of total nitrogen fluxes to the northern Gulf of Mexico. All results are reported as percentage changes in peak hypoxic area and volume relative to the baseline scenario with no carbon pricing mechanism and no widespread wetland restoration policy in place.

S.8 Inter-model sensitivities

Sensitivity of hypoxic area and volume to the cascade of model predictions are depicted in Figure 4 of the main text. Wetland denitrification is parameterized using an efficiency loss model (65) and we vary the constant in the log-log relationship between denitrification uptake velocity (m yr^{-1}) and nitrate concentration between typical (27 m y^{-1}) and high (54 m y^{-1}) values. Increasing the uptake velocity constant typically adds a further reduction of approximately 0.4% hypoxic area and 0.6% hypoxic volume across the suite of simulated scenarios when comparing to scenarios with the same assumptions other than the baseline uptake velocity value. There is smaller sensitivity to variation in the degree of fertilizer substitutability on the part of nitrate export, hypoxic area, or hypoxic volume, since the scope for reducing fertilizer use per unit of corn output is small. Most of the fertilizer reduction is due to a reduction in overall corn production (Figure 2) which depends on the overall demand for corn, both in the US and abroad. The fraction of nitrate entering deep subsurface storage on net also has only a small impact, with the three values simulated, producing variation in hypoxic area and volume of less than 0.1% in nearly all cases. Beyond the model parameters explored through our limited sensitivity analyses, each model makes a number of assumptions and relies on inputs from a suite of exogenous sources. Table S6 provides a summary of some of the most critical assumptions, inputs, and parameters and how they affect core outcomes of the modeling.

Table S6: Core assumptions, inputs, and parameters used throughout the suite of models. Processes or parameters in **bold** are varied in our sensitivity analysis.

Modeling assumption	Effect on ΔFertilizer	Effect on ΔNGMHZ
	ENVISAGE	
**SCC**	Higher/lower SCC is associated with higher/lower price of fertilizer and results in lower/higher fertilizer application rates calculated by SIMPLE-G-US-CS	Fertilizer application rates are linked to the amount of N reaching Gulf of Mexico as simulated by SIMPLE-G-US-CS, Agro-IBIS, and WBM.
CBAM	Implementation of the CBAM by the US prevents increasing imports of fertilizer from countries with less stringent mitigation policies. Non-implementation of CBAM would lead to increasing fertilizer imports, and higher application.	Non-implementation of the CBAM would result in higher amount of N reaching Gulf of Mexico
	Agro-IBIS	
Planting date and fertilization schedule based on meteorological input.	Spatial and temporal variability in farmer fertilization practice changes is not captured under different economic conditions.	Spatial and temporal differences in resulting nitrate leaching interacts with downstream denitrification processes in WBM.
N applied from inorganic fertilizer and manure treated similarly.	Potential for biases in amount and timing of nitrate leaving the root zone	Interactive biases with downstream denitrification processes in WBM.
	SIMPLE-G-US-CS	
**Grid-specific elasticity of substitution between nitrogen fertilizer and augmented land input**	More elastic substitution leads to a larger reduction in nitrogen fertilizer application for the same magnitude of nitrogen price shock. Alters total nitrate leached from croplands.	Change in nitrate leaching influences runoff and nitrate loading to the Gulf of Mexico through WBM.
Grid-specific cost shares of nitrogen fertilizer computed based on baseline year fertilizer application, fertilizer price, and commodity price.	Potentially higher fertilizer cost share would lead to a larger reduction in nitrogen application and potentially low bias in the predicted reductions in fertilizer application and leaching.	Change in nitrate leaching influences runoff and nitrate loading to the Gulf of Mexico through WBM.
**Level of adoption of a wetland restoration** intervention designed to treat nitrogen runoff from tile-drained corn/soy crops.	Minimal effect on fertilizer application because little land is removed from production when spillovers are taken into account.	Reduction in nitrate reaching stream and ultimately the Gulf of Mexico as simulated by WBM.
Wetlands occupy no more than 0.5% of their catchment.	Large wetland catchments both minimize land taken out of production and limit total areas that can be restored.	Limits to wetland catchment size and location limit the total nitrate reduction of this intervention.

Restored wetland catchments consist solely of corn/soy cropland.	Limiting catchments solely to corn/soy cropland maximizes nitrate removal per unit land removed.	Maximizes nitrate reduction at the Gulf of Mexico for the amount of land removed from corn/soy production.
	WBM	
**Wetland denitrification efficiency as the uptake velocity at the reference concentration (1 mg NO_3-N L^{-1}).**	N/A	Because new wetlands receive runoff from tile-drained corn-soy and input concentrations are high, a doubling of uptake velocity nearly doubles relative nitrate reductions from wetlands.
**Fraction of nitrate leachate entering (on net) deep groundwater / subsurface storage.**	N/A	Because leachate that leaves the surface hydrologic system is unavailable for other mitigation measures, relative changes in nitrate fluxes are inversely proportional to the magnitude of deep storage modeled.
All cropland leachate to wetland	N/A	Upper bound of nitrate leachate treated by wetlands.
TTDs are stationary in catchments.	N/A	Dynamic TTDs likely increase early season (May) nitrate export.
	Hypoxia	
Nitrites, ammonium, and dissolved organic nitrogen are relatively constant	N/A	Combined loads estimated at 44% of average nitrate loads from 1980 to 2021
Deliveries at mouth of Atchafalaya River assumed proportional to deliveries at mouth of Mississippi River	N/A	36% of Mississippi River value based on calibration data

NGMHZ – Northern Gulf of Mexico hypoxic zone
SCC – Social cost of carbon
CBAM – Country border adjustment measure
TTD – Transit time distribution

SI References

1. B. Storrow, Price hike marks new era for Calif. cap and trade. *E&E News* (2022) (September 13, 2022).

2. International Carbon Action Partner (ICAP), ICAP Allowance Price Explorer (2022) (September 12, 2022).

3. , Allowance Prices and Volumes | RGGI, Inc. (2022) (September 12, 2022).

4. U.S. Energy Information Administration, "Reducing Greenhouse Gases in the United States: A 2030 Emissions Target" (2022) (September 12, 2022).

5. P. S. Armington, A Theory of Demand for Products Distinguished by Place of Production (Une théorie de la demande de produits différenciés d'après leur origine) (Una teoría de la demanda de productos distinguiéndolos según el lugar de producción). *Staff Papers (International Monetary Fund)* **16**, 159–178 (1969).

6. M. Chepeliev, GTAP- Power Database: Version 10. *JGEA* **5**, 110–137 (2020).

7. J. Caron, Estimating carbon leakage and the efficiency of border adjustments in general equilibrium — Does sectoral aggregation matter? *Energy Economics* **34**, S111–S126 (2012).

8. T. Hertel, D. van der Mensbrugghe, Chapter 14: Behavioral Parameters. *Center for Global Trade Analysis* (2016).

9. EMBER, Carbon Price Tracker. THe price of emissions allowances in EU and UK (2023).

10. M. Chepeliev, Possible Implications of the European Carbon Border Adjustment Mechanism for Ukraine and Other EU Trading Partners. **2** (2021).

11. , Technical Support Document: Social Cost of Carbon, Methane, and Nitrous Oxide (2021).

12. S. Dröge, *et al.*, "Tackling Leakage in a World of Unequal Carbon Prices" (Climate Strategies, 2009) (July 14, 2023).

13. K. Feng, S. J. Davis, L. Sun, K. Hubacek, Drivers of the US CO2 emissions 1997–2013. *Nat Commun* **6**, 7714 (2015).

14. S. Liang, *et al.*, Socioeconomic Drivers of Greenhouse Gas Emissions in the United States. *Environ. Sci. Technol.* **50**, 7535–7545 (2016).

15. International Energy Agency, "Global Energy Review: CO2 Emissions in 2021 – Analysis" (IEA, 2023) (July 14, 2023).

16. S. Menegat, A. Ledo, R. Tirado, Greenhouse gas emissions from global production and use of nitrogen synthetic fertilisers in agriculture. *Sci Rep* **12**, 14490 (2022).

17. S. A. Nosherwani, R. C. Neto, Techno-economic assessment of commercial ammonia synthesis methods in coastal areas of Germany. *Journal of Energy Storage* **34**, 102201 (2021).

18. N. Campion, H. Nami, P. R. Swisher, P. Vang Hendriksen, M. Münster, Techno-economic assessment of green ammonia production with different wind and solar potentials. *Renewable and Sustainable Energy Reviews* **173**, 113057 (2023).

19. International Energy Agency, "Ammonia Technology Roadmap. Towards more sustainable nitrogen fertilizer production." (IEA, 2021).

20. International Energy Agency, "Energy Technology Perspectives 2023" (IEA, 2023).

21. T. A. Maung, D. G. Ripplinger, G. J. McKee, D. M. Saxowsky, Eds., *Economics of Using Flared vs. Conventional Natural Gas to Produce Nitrogen Fertilizer: A Feasibility Analysis* (2012) https:/doi.org/10.22004/ag.econ.133410.

22. S. Cloete, M. N. Khan, S. M. Nazir, S. Amini, Cost-effective clean ammonia production using membrane-assisted autothermal reforming. *Chemical Engineering Journal* **404**, 126550 (2021).

23. International Energy Agency, "Indicative production costs for ammonia via electrolysis in selected regions compared to current references." (IEA, 2023).

24. C. J. Kucharik, Evaluation of a Process-Based Agro-Ecosystem Model (Agro-IBIS) across the U.S. Corn Belt: Simulations of the Interannual Variability in Maize Yield. *Earth Interactions* **7**, 1–33 (2003).

25. C. J. Kucharik, K. R. Brye, Integrated BIosphere Simulator (IBIS) Yield and Nitrate Loss Predictions for Wisconsin Maize Receiving Varied Amounts of Nitrogen Fertilizer. *Journal of Environmental Quality* **32**, 247–268 (2003).

26. S. D. Donner, C. J. Kucharik, Corn-based ethanol production compromises goal of reducing nitrogen export by the Mississippi River. *Proceedings of the National Academy of Sciences* **105**, 4513–4518 (2008).

27. G. D. Farquhar, S. Von Caemmerer, J. A. Berry, A biochemical model of photosynthetic CO2 assimilation in leaves of C3 species. *Planta* **149**, 78–90 (1980).

28. G. J. Collatz, J. T. Ball, C. Grivet, J. A. Berry, Physiological and environmental regulation of stomatal conductance, photosynthesis and transpiration: a model that includes a laminar boundary layer. *Agricultural and Forest Meteorology* **54**, 107–136 (1991).

29. S. L. Thompson, D. Pollard, A Global Climate Model (GENESIS) with a Land-Surface Transfer Scheme (LSX). Part I: Present Climate Simulation. *Journal of Climate* **8**, 732–761 (1995).

30. S. L. Thompson, D. Pollard, A Global Climate Model (GENESIS) with a Land-Surface Transfer Scheme (LSX). Part II: CO2 Sensitivity. *Journal of Climate* **8**, 1104–1121 (1995).

31. C. Kucharik, A. VanLoocke, J. Lenters, M. Motew, Miscanthus Establishment and Overwintering in the Midwest USA: A Regional Modeling Study of Crop Residue Management on Critical Minimum Soil Temperatures. *Papers in Natural Resources* (2013).

32. M. M. Motew, C. J. Kucharik, Climate-induced changes in biome distribution, NPP, and hydrology in the Upper Midwest U.S.: A case study for potential vegetation: CLIMATE IMPACTS IN THE UPPER MIDWEST U.S. *J. Geophys. Res. Biogeosci.* **118**, 248–264 (2013).

33. J. Liu, *et al.*, Multi-scale Analysis of Nitrogen Loss Mitigation in the US Corn Belt (2022) https:/doi.org/10.48550/arXiv.2206.07596 (September 12, 2022).

34. D. A. Miller, R. A. White, A Conterminous United States Multilayer Soil Characteristics Dataset for Regional Climate and Hydrology Modeling. *Earth Interact.* **2**, 1–26 (1998).

35. C. R. Schwalm, *et al.*, A model-data intercomparison of CO_2 exchange across North America: Results from the North American Carbon Program site synthesis. *J. Geophys. Res.* **115**, G00H05 (2010).

36. M. C. Dietze, *et al.*, Characterizing the performance of ecosystem models across time scales: A spectral analysis of the North American Carbon Program site-level synthesis. *J. Geophys. Res.* **116**, G04029 (2011).

37. K. Schaefer, *et al.*, A model-data comparison of gross primary productivity: Results from the North American Carbon Program site synthesis: A MODEL-DATA COMPARISON OF GPP. *J. Geophys. Res.* **117**, n/a-n/a (2012).

38. P. C. Stoy, *et al.*, Evaluating the agreement between measurements and models of net ecosystem exchange at different times and timescales using wavelet coherence: an example using data from the North American Carbon Program Site-Level Interim Synthesis. *Biogeosciences* **10**, 6893–6909 (2013).

39. S. D. Donner, C. J. Kucharik, Evaluating the impacts of land management and climate variability on crop production and nitrate export across the Upper Mississippi Basin. *Global Biogeochem. Cycles* **17**, n/a-n/a (2003).

40. C. J. Kucharik, *et al.*, A multiyear evaluation of a Dynamic Global Vegetation Model at three AmeriFlux forest sites: Vegetation structure, phenology, soil

temperature, and CO2 and H2O vapor exchange. *Ecological Modelling* **196**, 1–31 (2006).

41. C. J. Kucharik, T. E. Twine, Residue, respiration, and residuals: Evaluation of a dynamic agroecosystem model using eddy flux measurements and biometric data. *Agricultural and Forest Meteorology* **146**, 134–158 (2007).

42. T. E. Twine, C. J. Kucharik, Evaluating a terrestrial ecosystem model with satellite information of greenness. *J. Geophys. Res.* **113**, G03027 (2008).

43. M. E. Soylu, C. J. Kucharik, S. P. Loheide, Influence of groundwater on plant water use and productivity: Development of an integrated ecosystem – Variably saturated soil water flow model. *Agricultural and Forest Meteorology* **189–190**, 198–210 (2014).

44. M. Motew, *et al.*, The Influence of Legacy P on Lake Water Quality in a Midwestern Agricultural Watershed. *Ecosystems* **20**, 1468–1482 (2017).

45. U. L. C. Baldos, I. Haqiqi, T. Hertel, M. Horridge, J. Liu, SIMPLE-G: A multiscale framework for integration of economic and biophysical determinants of sustainability. *Environmental Modelling & Software*, 104805 (2020).

46. S. Sun, *et al.*, Fine-Scale Analysis of the Energy–Land–Water Nexus: Nitrate Leaching Implications of Biomass Cofiring in the Midwestern United States. *Environ. Sci. Technol.* **54**, 2122–2132 (2020).

47. T. J. Lark, *et al.*, Environmental outcomes of the US Renewable Fuel Standard. *Proceedings of the National Academy of Sciences* **119**, e2101084119 (2022).

48. C. E. Ferguson, *The neoclassical theory of production and distribution* (Cambridge University Press, 2008).

49. J. w. Gilliam, J. l. Baker, K. r. Reddy, “Water Quality Effects of Drainage in Humid Regions” in *Agricultural Drainage*, (John Wiley & Sons, Ltd, 1999), pp. 801–830.

50. E. Marshall, “Reducing Nutrient Losses From Cropland in the Mississippi/Atchafalaya River Basin: Cost Efficiency and Regional Distribution” (US Department of Agiculture, Economic Research Service, 2018).

51. L. Christianson, J. Tyndall, M. Helmers, Financial comparison of seven nitrate reduction strategies for Midwestern agricultural drainage. *Water Resources and Economics* **2–3**, 30–56 (2013).

52. F. T. Portmann, Siebert Stefan, Döll Petra, MIRCA2000—Global monthly irrigated and rainfed crop areas around the year 2000: A new high-resolution data set for agricultural and hydrological modeling. *Global Biogeochemical Cycles* **24** (2010).

53. N. Ramankutty, A. T. Evan, C. Monfreda, J. A. Foley, Farming the planet: 1. Geographic distribution of global agricultural lands in the year 2000. *Global Biogeochemical Cycles* **22** (2008).

54. C. Monfreda, N. Ramankutty, J. A. Foley, Farming the planet: 2. Geographic distribution of crop areas, yields, physiological types, and net primary production in the year 2000. *Global Biogeochemical Cycles* **22** (2008).

55. T. J. Lark, J. M. Salmon, H. K. Gibbs, Cropland expansion outpaces agricultural and biofuel policies in the United States. *Environ. Res. Lett.* **10**, 044003 (2015).

56. D. Shrestha, K. Masarik, C. J. Kucharik, Nitrate losses from Midwest US agroecosystems: Impacts of varied management and precipitation. *Journal of Soil and Water Conservation* **78**, 141–153 (2023).

57. R. Stewart, *et al.*, Separation of River Network-Scale Nitrogen Removal among the Main Channel and Two Transient Storage Compartments. *Water Resources Research - WATER RESOUR RES* **47** (2011).

58. W. M. Wollheim, B. J. Peterson, S. M. Thomas, C. H. Hopkinson, C. J. Vörösmarty, Dynamics of N removal over annual time periods in a suburban river network. *Journal of Geophysical Research: Biogeosciences* **113** (2008).

59. N. R. Samal, *et al.*, A coupled terrestrial and aquatic biogeophysical model of the Upper Merrimack River watershed, New Hampshire, to inform ecosystem services evaluation and management under climate and land-cover change. *Ecology and Society* **22**, 18 (2017).

60. D. S. Grogan, *et al.*, Water balance model (WBM) v.1.0.0: a scalable gridded global hydrologic model with water-tracking functionality. *Geoscientific Model Development* **15**, 7287–7323 (2022).

61. S. Zuidema, W. Wollheim, C. Kucharik, R. Lammers, Existing wetland conservation programs miss nutrient reduction targets. *Nature Communications* (2022) https:/doi.org/10.21203/rs.3.rs-2386822/v1 (January 3, 2023).

62. D. Yamazaki, *et al.*, MERIT Hydro: A High-Resolution Global Hydrography Map Based on Latest Topography Dataset. *Water Resources Research* **55**, 5053–5073 (2019).

63. D. Scavia, M. A. Evans, D. R. Obenour, A Scenario and Forecast Model for Gulf of Mexico Hypoxic Area and Volume. *Environ. Sci. Technol.* **47**, 10423–10428 (2013).

64. S. P. Seitzinger, *et al.*, Global river nutrient export: A scenario analysis of past and future trends. *Global Biogeochemical Cycles* **24** (2010).

65. W. M. Wollheim, *et al.*, Nitrate uptake dynamics of surface transient storage in stream channels and fluvial wetlands. *Biogeochemistry* **120**, 239–257 (2014).
